# Identification of protein structural elements responsible for the diversity of sequence preferences among Mini-III RNases

**DOI:** 10.1038/srep38612

**Published:** 2016-12-07

**Authors:** Dawid Głów, Małgorzata Kurkowska, Justyna Czarnecka, Krzysztof Szczepaniak, Dariusz Pianka, Verena Kappert, Janusz M. Bujnicki, Krzysztof J. Skowronek

**Affiliations:** 1 Laboratory of Bioinformatics and Protein Engineering, International Institute of Molecular and Cell Biology in Warsaw, ul. Ks. Trojdena 4, 02-109 Warsaw, Poland

## Abstract

Many known endoribonucleases select their substrates based on the presence of one or a few specific nucleotides at or near the cleavage site. In some cases, selectivity is also determined by the structural features of the substrate. We recently described the sequence-specific cleavage of double-stranded RNA by Mini-III RNase from *Bacillus subtilis in vitro*. Here, we characterized the sequence specificity of eight other members of the Mini-III RNase family from different bacterial species. High-throughput analysis of the cleavage products of Φ6 bacteriophage dsRNA indicated subtle differences in sequence preference between these RNases, which were confirmed and characterized by systematic analysis of the cleavage kinetics of a set of short dsRNA substrates. We also showed that the sequence specificities of Mini-III RNases are not reflected by different binding affinities for cognate and non-cognate sequences, suggesting that target selection occurs predominantly at the cleavage step. We were able to identify two structural elements, the α4 helix and α5b-α6 loop that were involved in target selection. Characterization of the sequence specificity of the eight Mini-III RNases may provide a basis for better understanding RNA substrate recognition by Mini-III RNases and adopting these enzymes and their engineered derivatives as tools for RNA research.

Studies on enzymes that are involved in RNA metabolism are highly important for the field of RNA biology. In addition to uncovering the molecular mechanisms of RNA processing, they can provide novel tools for RNA research. Advances in RNA studies are hampered by the lack of key enzymatic tools, the counterparts of which are available for DNA and protein research. The availability of sequence-specific endoribonucleases (endoRNases) would enable easy, reproducible and controlled fragmentation of RNA molecules both *in vivo* and *in vitro.*

Many endoRNases that act on single stranded RNA exhibit some sequence preference, but their target sites are usually limited to one or a few specific nucleotides. Additionally, cleavage frequently depends on the structure of the substrate. Examples include the following prokaryotic single strand specific endoRNases: T1, which cleaves 3′ to guanosine residues with high specificity^1^, RegB, which cleaves its mRNA substrates in the middle of the GGAG sequence^2^ if the last G is located in a short stem between two loops^3^, and α–sarcin RNase, which cleaves 28 S rRNA in the GAGA sequence within a loop^4^.

Recently, successful attempts were described to engineer sequence specific single-stranded RNases, leading to the development of novel enzymes with narrow, well-defined sequence specificities, e.g., fusion of RNase T1 and the TAT peptide^5^ and fusion of a PIN nuclease with a PUF domain^6^. Other recent developments involve hammerhead ribozymes^7^, catalytically active DNA molecules (DNAzymes)^8^, peptide nucleic acid-based artificial nucleases (PNAzymes)^9^ and Cas9 nuclease^10^, which have been engineered to enable the site-specific cleavage of RNA molecules.

Until recently, no endoRNase with sequence specificity that is comparable to restriction enzymes has been described for double-stranded RNA (dsRNA). The best studied endoRNase that cuts dsRNA is bacterial RNase III. A conserved catalytic domain of RNase III is present in a large group of enzymes that comprise the RNase III superfamily. The classification of RNase III enzymes into subfamilies is based on the presence of various functional elements, in addition to the catalytic domain^11^. Class 1 of the RNase III group of enzymes (i.e., classical RNase III) consists of an RNase III domain and dsRNA binding domain (dsRBD) and acts as homodimers. Class 2 and class 3 enzymes are represented by Drosha and Dicer, respectively. They contain two RNase III domains and a single dsRBD and usually act as monomers. Class 2 enzymes also possess a polyproline domain. Class 3 enzymes usually have three additional domains: DExD helicase, DUF283, and PAZ. Class 4 proteins, called Mini-III, are the smallest members of the RNase III superfamily. They consist solely of a catalytic domain and act as homodimers.

Determination of the crystal structure of a canonical RNase III in complex with dsRNA revealed patterns of interactions between the enzyme and substrate that involve four regions, so called RNA binding motifs (RBM)^12^. Two RBMs are located within the catalytic domain, and two are within the dsRBD. The results of biochemical studies indicated that the structural features of the RNA substrate play a major role in determining the preferred cleavage site of classical RNase III^13^. Analysis of the effects of different substitutions that were introduced in close proximity to the RNase III cleavage site revealed the existence of a weak consensus sequence (WNAGWGNNCWUNNN^NAWGNNCWCUNW, where W = A or U, N = any residue, and ^ = a scissile phosphodiester bond)^14^.

Mini-III RNases are found in Gram-positive bacteria and plastids of plants. *Bacillus subtilis* Mini-III (BsMiniIII) is involved in the last step of 23 S rRNA maturation, i.e., the final cleavage of pre-rRNA^11^. *In vitro* reactivity on the pre-rRNA substrate strongly depends on the ribosomal protein L3, which likely facilitates recognition of the cleavage site in this substrate^15^. In plastids, Mini-III is involved in ribosomal RNA maturation and spliced intron degradation^16^. Members of class 4 do not possess dsRBD; thus, the mechanism of the substrate recognition process must differ from canonical RNase III and remains unclear^15^. Another difference is the substitution of a long loop α5-α6 in canonical RNase III with a helix (α5b) and short loop that connects the α5b and α6 helices.

We previously reported that BsMiniIII was able to cleave long dsRNA substrates, as a standalone sequence-specific dsRNase, in addition to its previously known ability to cleave, in complex with the L3 protein, the irregular structure in pre-rRNA. We also defined the cleavage site sequence (ACC^U, where ^ = a scissile phosphodiester bond) preferred by BsMiniIII. We showed that the loop α5b-α6 that is present in Mini-III proteins and absent in classical RNase III enzymes is indispensable for specific dsRNA cleavage by BsMiniIII, but it is not required for dsRNA binding^17^. Here, we characterize the sequence preferences of eight other members of the Mini-III family. We confirmed the involvement of the loop α5b-α6 in cleavage site selection by these enzymes and found that the helix α4 also participates in this process. Both elements have also impact on cleavage rates.

## Results

### Characterization of the limited cleavage of the Φ6 RNA by Mini-III RNases

**Figure 1:**
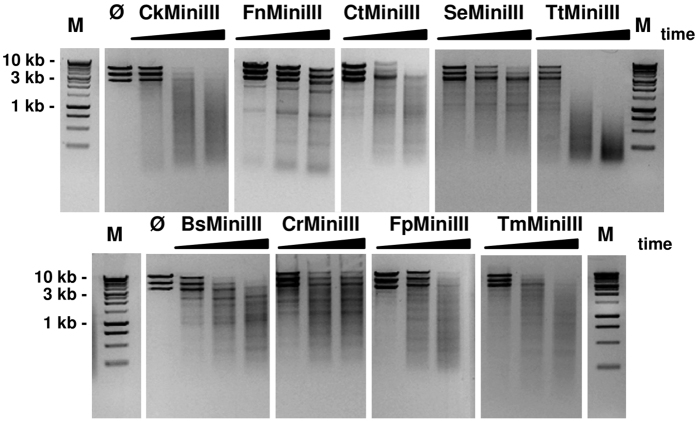
Cleavage patterns of the Φ6 genomic RNA by Mini-III enzymes. dsRNA (1.5 μg) was digested with 3.3 μg BsMiniII, 80 ng of CkMiniIII, 23.5 μg of CrMiniIII, 5 μg of CtMiniIII, 0.8 μg of FnMiniIII, 1.1 μg of FpMiniIII, 2.1 μg of SeMiniIII, 0.185 μg of TmMiniIII, and 11.5 ng of TtMiniIII under favorable conditions (see Table 1) for each enzyme. Aliquots were taken at 5, 10, and 15 min, with the exception of TtMiniIII, where aliquots were taken at 2, 4, and 6 min, and SeMiniIII, in which aliquots were taken at 20, 40, and 60 min. Ø indicates an untreated substrate. M - dsDNA molecular weight marker.

In our previous study^17^, we reported evidence of the sequence-dependent cleavage of long dsRNA molecules and characterized the sequence preference of BsMiniIII. Simple sequence database searches identified more than 600 members of class 4 RNase III enzymes. We selected eight proteins with diverse amino acid sequences in the loop α5b-α6 and we tested their cleavage specificity using the Φ6 bacteriophage dsRNA as a substrate (Table 1, Supplementary Table S1, Supplementary Fig. S1). The band patterns that were generated by different enzymes were clearly discernible and apparently different from the pattern generated by the previously characterized BsMiniIII enzyme (Fig. 1). *In vitro* cleavage of the 23 S pre-rRNA, a natural substrate of BsMiniIII is substantially increased in the presence of the ribosomal protein L3 15. In contrast, BsMiniIII cleavage of the Φ6 dsRNA in the presence of the *B. subtilis* L3 protein is slightly decreased and no change in the band pattern is observed, which suggests that L3 has no influence on the cleavage site selection in long dsRNA (Supplementary Fig. S2).

### Characterization of the sequence preference based on high-throughput sequencing

**Figure 2:**
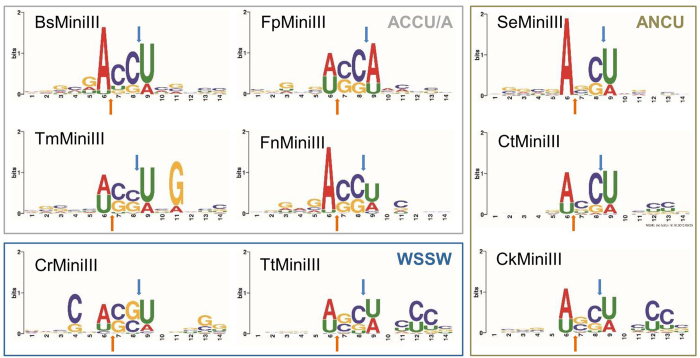
Sequence profiles of the preferred cleavage sites of Mini-III enzymes based on high-throughput sequencing. Blue arrows indicate cleavage sites in the sequences shown; orange arrows indicate cleavage sites in the complementary strands.

To determine the quantitative characteristics of the cleavage preference of the Mini-III family members, we applied high-throughput sequencing of the ends that were generated in the time-limited cleavage reactions of Φ6 dsRNA. For each enzyme we analyzed between 175,000 and 376,000 reads, that passed quality filter and were mapped to the Φ6 genome. In the control experiment, in which Φ6 dsRNA was incubated in the Bs reaction buffer for 5 min at 37 °C in the absence of any enzyme and then processed similarly to the other samples, 93% of the reads started from the physical ends of the genomic segments. This confirmed that the internal reads that were obtained for dsRNA after cleavage were almost certainly genuine cleavage products and not artifacts of the preparation method. 200 14-base-pair sequences, each representing one of 200 of the most frequently observed cleavage sites, were used to build sequence profiles for each Mini-III enzyme, from which sequence logos and consensus sequences were inferred (Fig. 2). For the majority of the enzymes, the consensus sequences could be summarized as WSSW (where S = G or C and W = A or U). Nevertheless, in agreement with the gel analysis of the band patterns after limited cleavage of the same substrate (Φ6 dsRNA), clear differences could be seen between consensus sequences of the preferred cleavage sites for the enzymes analyzed herein. BsMiniIII appeared to be the most specific, with a clear preference for the ACCU sequence. CtMiniIII and SeMiniIII appeared to be less specific for position 7 of the sequence logo, which could be occupied by any nucleotide. For FnMiniIII, the consensus was less stringent for the central dinucleotide, and positions 7 and 8 in the cleavage site (numbering according to the 14-nt sequence logo) could be occupied by either C or G. For FpMiniIII and TmMiniIII, also position 6 appeared to be less stringently discriminated as it could be occupied by either A or U. CkMiniIII, CrMiniIII, and TtMiniIII appeared to be the least specific among the enzymes analyzed herein, and their consensus sequences suggested that any variation of a WSSW motif could be efficiently cleaved.

High-throughput sequencing results also indicated that some of the Mini-III enzymes discriminated between substrates depending on sequence positions outside the tetranucleotide core. The most apparent was the preference of CrMiniIII and TmMiniIII for C in position 4 and for G in position 11, respectively. Moreover, for CkMiniIII and TtMiniIII, the preference for C in positions 12 and 13 was detected.

To relate observed differences in sequence preferences among Mini-III enzymes to differences in their natural substrates, we aligned 23 S pre-rRNA sequences from bacterial hosts studied in this work and analyzed sequence conservation and predicted structure in the region homologous to the known cleavage site of BsMiniIII^11^ (Supplementary Fig. S3).

For all microorganisms we were able to find sequences corresponding to the sequence profile of the preferred cleavage sites in the double stranded stems of the 23 S pre-rRNA. In five out of eight cases the location of the predicted cleavage site is similar to that in *B. subtilis*, i.e. at the end of the stem. In *T. maritima* 23 S pre-rRNA the predicted helical region is extended by three additional base pairs in such a way that the cleavage site may be shifted in relation to *B. subtilis* 23 S pre-rRNA. In *F. nucleatum* 23 S pre-rRNA the potential cleavage site (AGGU/AUCU) contains a wobble pair U • G and is present within the dsRNA region. Finally, in *C. ramnosum* 23 S pre-rRNA the region under consideration with the AGGU/ACCU site has a very weak propensity to form a helical structure, and the 3′ segment exhibits much stronger tendency to pair with another region further downstream. The predicted cleavage site is located within short dsRNA region.

To validate the results of the high-throughput analysis, we prepared five short fragments of the Φ6 genome that encompassed single cleavage sites. According to the sequencing results, these substrates were cleaved with high frequency, and we used them as substrates for *in vitro* cleavage assays (Supplementary Table S2, Supplementary Fig. S4). The sizes of the major reaction products agreed with cleavage within sites found in the high-throughput data. Additionally, the relative cleavage efficiencies of the selected substrates agreed with the relative cleavage frequencies that were observed in the sequencing results. For example, the 949 S substrate was cleaved efficiently only by FpMiniIII, for which it was highly scored in the high-throughput data, whereas other enzymes, for which this position was not found among the most frequent sequencing reads, did not cleave it at all or cleaved it barely (FnMiniIII). Secondary cleavage sites (i.e., sites responsible for generation of additional bands) were found in several cases; see BsMiniIII cleavage of 4486 L substrate; CrMiniIII cleavage of 3292 L substrate or FnMiniIII cleavage of 2021 L substrate in Supplementary Fig. S4.They may be explained by the additional cleavage events that were observed in the high-throughput data, however with frequency much lower than the primary cleavage sites. Consistent with the differences that were visible in the Φ6 cleavage patterns and in consensus sequences of the preferred cleavage sites derived from high-throughput sequencing, these results indicated differences in the sequence preferences of some of the enzymes.

### Effect of base-pair substitutions on the substrate cleavage efficiency

#### Substitutions in the central tetranucleotide

**Figure 3:**
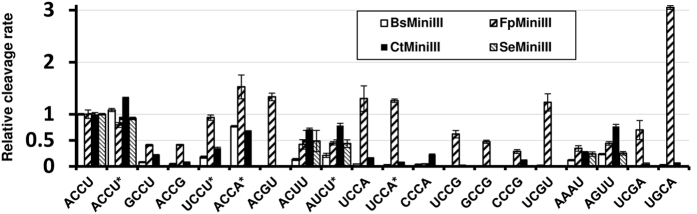
Effects of substitutions introduced into the cleavage site of substrate 910S-ACCU on cleavage rates. For each enzyme, cleavage rates were normalized to the rate measured for the original 910S-ACCU substrate. *The sequence of the complementary strand is shown.

To further investigate the differences between the Mini-III enzymes in a systematic way we analyzed the effects of substitutions in the ACCU sequence of the 910 S substrate on the cleavage activity of four enzymes: BsMiniIII, CtMiniIII, FpMiniIII, and SeMiniIII as they represent well the diversity of the cleavage consensus sequences (Fig. 2). Nineteen variants of the substrate that were obtained by site-directed mutagenesis were used to study the single-turnover kinetics of selected Mini-III enzymes (Fig. 3, Supplementary Fig. S5). The only derivative of the 910 S substrate that was cleaved by BsMiniIII, CtMiniIII, and SeMiniIII with rates similar to the original substrate was one reversely complementary to the ACCU sequence. SeMiniIII was unable to cleave any other substrate variant with efficiency greater than 50% of the efficiency that was measured for the 910S-ACCU substrate. BsMiniIII cleaved only the ACCA variant with efficiency that was greater than 50% of the efficiency that was measured for the wild type substrate. For CtMiniIII, ACCA, ACUU, AUCU, and AGUU were also cleaved with efficiency that was greater than 50% of the efficiency that was measured for the wild type substrate. For FpMiniIII, the best cleaved substrate contained the UGCA sequence and was cleaved threefold faster than the original sequence. Furthermore, FpMiniIII cleaved substrates that contained UCCU, ACCA, ACGU, UCCA, and UCGU sequences with efficiencies similar to or greater than the cleavage of its original substrate. Results of this experiment showed that all deviations from the ACCU consensus sequence in the cleavage site resulted in noticeable decrease of the cleavage rate, with the exception of the least specific FpMiniIII.

#### Substitutions outside the central tetranucleotide

We next tested the importance of the positions 3, 4, 11 and 12 (i.e., outside the central tetranucleotide) by studying the effects of substitutions at these positions on the cleavage reactivity (Supplementary Fig. S6). These effects did not always agree with the predictions based on consensus sequences obtained from high-throughput sequencing experiments. For instance, TtMiniIII was able to cut all substrates tested, including those with C to A substitution at position 12, which violated the consensus sequence for this enzyme (Fig. 2) calculated from high-throughput cleavage experiments.

CrMiniIII (consensus sequence with C being the dominant residue at position 4; Fig. 2) was able to cleave the 910S-ACCU substrate with A at position 4. Surprisingly, the A4C substitution in this substrate that made it more “consensus-like” decreased the cleavage efficiency of CrMiniIII. A similar decrease was seen also for the A4U substitution in this substrate. The only residue that was well tolerated by CrMiniIII at position 4 was G (seen also in the consensus as the second most common residue at position 4). Hence, tests of 910S-ACCU substrate variants indicated the preference of CrMiniIII for A or G at position 4, rather than C and G observed in high-throughput experiments. The other results were more consistent with the high-throughput sequencing results. Consistent with these data, CkMiniIII was mostly impaired by the C12A substitution in the 910S-ACCU substrate, however it was also affected by a A4C substitution, which could not be predicted from high-throughput data. TmMiniIII activity on 910S-ACCU substrate variants agreed with the predictions from high throughput data concerning the preference for G at position 11, and additionally indicated a relative preference for G over U at position 3, C over A in position 4, and C over A at position 12.

The general conclusion from above-mentioned experiments is that effects of substitutions at positions 3, 4, 11 and 12 in the 910S-ACCU substrate, similarly to the effects of substitutions in the central tetranucleotide (positions 6-9) generally agreed with predictions of sequence preferences of individual enzymes inferred from the high-throughput sequencing results. These results also confirmed that there are differences in sequence preferences of the Mini-III enzymes.

### Effects of swapping the α4 helix and the α5b-α6 loop on activity and specificity of Mini-III enzymes

**Figure 4:**
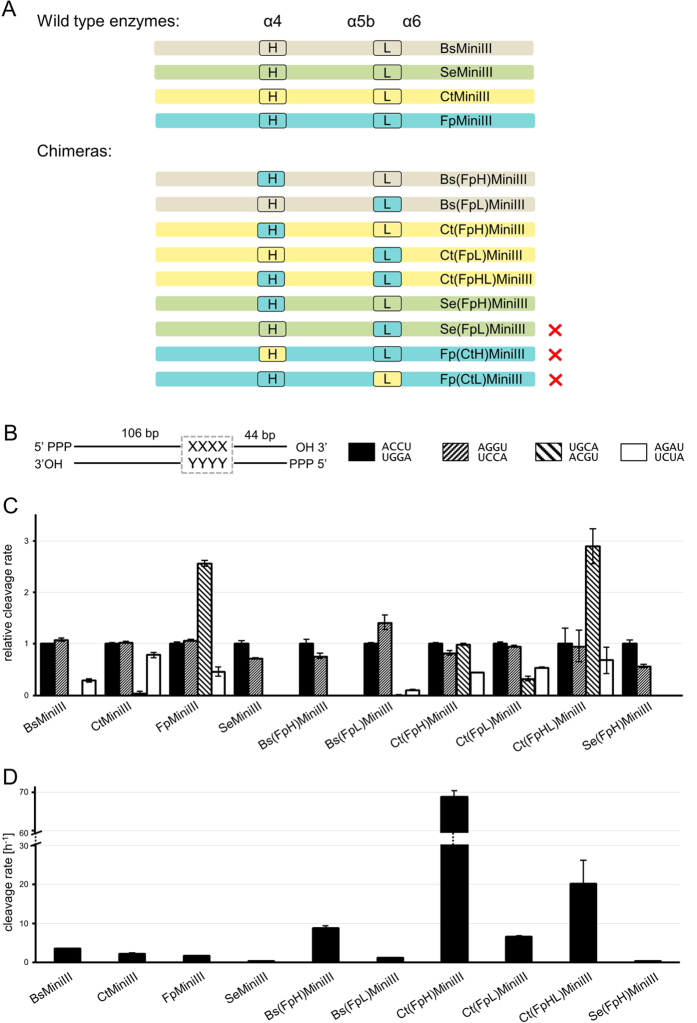
Effects of swapping the α4 helix and α5b-α6 loop in chimeric Mini-III enzymes. (**A**) Schematic representation of the chimeric Mini-III constructs. Red X signs on the right hand side indicate chimeras that were unable to cleave dsRNA. (**B**) Schematic representation of the substrate. (**C**) Cleavage sequence preference. For each enzyme, cleavage activity was normalized to the activity on the 910S-ACCU substrate. (**D**) Cleavage activity assayed on the 910S-ACCU substrate.

The results of our previous study^17^ suggested that the α5b-α6 loop of BsMiniIII is involved in the recognition and selection of the target sequence in dsRNA. According to the structural model of the Mini-III-dsRNA complex, in addition to this loop, the α4 helix is also in close proximity to the sequence cleaved (Supplementary Fig. S7). In the absence of a high-resolution crystal structure of the protein-RNA complex, which could verify the putative involvement of these structural elements in RNA sequence recognition, we used biochemical experiments that consisted of swapping amino acid sequences in one or the other of these two regions between related Mini-III enzymes, followed by assaying cleavage activities of such chimeric proteins. To guide the planning of such swaps and construction of chimeric proteins, we used sequence alignment of Mini-III enzymes (Supplementary Fig. S7). We selected conserved amino acid residues L45 and V53 as the boundaries of α4 helix swaps and residues N84 and Y99 as the boundaries for α5b-α6 loop swaps (the numbering refers to the native BsMiniIII sequence). We assayed the endoribonucleolytic activity of chimeric proteins that consisted of an acceptor Mini-III protein scaffold and one or two structural elements that were derived from another protein. As a substrate for activity assays, we used 910S RNA (ACCU target sequence) and its three selected substitution variants, with the target changed to AGGU, UGCA, or AUCU (Fig. 4). The nomenclature of the chimeric proteins that were obtained indicates (in parentheses) the source and identity of the swapped element. For example, CtMiniIII with part of the α4 helix from FpMiniIII was named Ct(FpH)MiniIII (Fig. 4A). Initially we constructed four chimeric proteins: Fp(CtH)MiniIII, Fp(CtL)MiniIII, Ct(FpH)MiniIII and Ct(FpL)MiniIII. Both constructs based on the FpMiniIII scaffold and with CtMiniIII “grafts” were inactive, whereas the reverse chimeras (CtMiniIII scaffold with “grafts” from FpMiniIII) were active. We also constructed chimeric proteins where the same fragments of FpMiniIII were grafted into scaffolds of BsMiniIII and SeMiniIII. One of these constructs, Se(FpL)MiniIII, was inactive. To test if the chimeric proteins are folded properly we measured FT-IR spectra of native Mini-III enzymes and their swapped variants. No apparent differences in shapes of amide I peak that would indicate significant differences in secondary structures content were visible suggesting that none of the chimeric proteins was misfolded (Supplementary Fig. S8, Supplementary Table S3). The Bs(FpL)MiniIII variant retained 34% of the activity of the wild type BsMiniIII enzyme, and the Se(FpH)MiniIII variant had approximately the same activity as the wild type SeMiniIII enzyme. In contrast, three variants in which FpMiniIII fragments were introduced into the CtMiniIII scaffold Ct(FpH)MiniIII, Ct(FpL)MiniIII and Ct(FpHL)MiniIII, as well as the Bs(FpH)MiniIII variant, all had much greater activity than the original enzymes. For instance, the activity of the Ct(FpH)MiniIII chimeric enzyme increased more than 30-fold compared to CtMiniIII or FpMiniIII (Fig. 4D).

In the case of chimeras with the CtMiniIII scaffold and FtMiniIII “grafts”, changes in cleavage preferences were observed. These changes were not as striking as in the case of the enzymatic activity, but were clearly visible. The 910S-UGCA RNA was a very poor substrate for CtMiniIII cleavage. Ct(FpH)MiniIII cleaved this substrate at a rate that was similar to that of the 910S-ACCU substrate. The Ct(FpHL)MiniIII variant cleaved 910S-UGCA threefold faster than the original 910S-ACCU substrate, similarly to the donor enzyme FpMiniIII. No changes in specificity were seen for any variant with the BsMiniIII or SeMiniIII scaffold (Fig. 4C).

The results of swapping experiments showed that a swap of the α4 helix and/or the α5b-α6 loop between different Mini-III proteins led in several cases to a substantial increase of enzymatic activity and alteration of sequence preferences. In the double chimera Ct(FpHL)MiniIII, where both the α5b-α6 loop and the α4 helix were swapped, the sequence preference was changed from that of the “acceptor” to that of the “donor” protein.

### Multiple turnover assay of the dsRNA cleavage

To test sequence preference of Mini-III enzymes on relatively large dsRNA substrates, which present many potential cleavage sites, including sites that deviate from the consensus and represent poorer targets, we used single turnover cleavage assays described above. Such assays provide important insight into cleavage site selection at the endonucleolytic step of the enzymatic reaction, while single turnover experiments neglect the product release step, which for many endonucleases is a rate-limiting in the enzymatic cycle. Multiple turnover cleavage measurements were also performed on two 28 bp dsRNAs (Supplementary Table S6), one that contained the preferred cleavage site from the 910 S substrate (POS-ACCU) and another one with the same nucleotide composition, but derived from a different fragment of 910 S sequence, in which no cleavage could be detected (NEG). The NEG substrate is therefore “scrambled”, uncleavable derivative of POS-ACCU. The POS-ACCU substrate was cleaved by all enzymes, and they all generated unique products (Supplementary Fig. S9). Only TmMiniIII and CkMiniIII cleaved the NEG substrate, although with significantly lower efficiency than the POS-ACCU substrate, and with no retention of discrete products. These results lead us to conclude that Mini-III RNases maintain sequence preference also under multiple turnover conditions. There is also qualitative correspondence between relative activities of the Mini-III enzymes in multiple turnover and in single turnover assays (Fig. 1).

**Figure 5:**
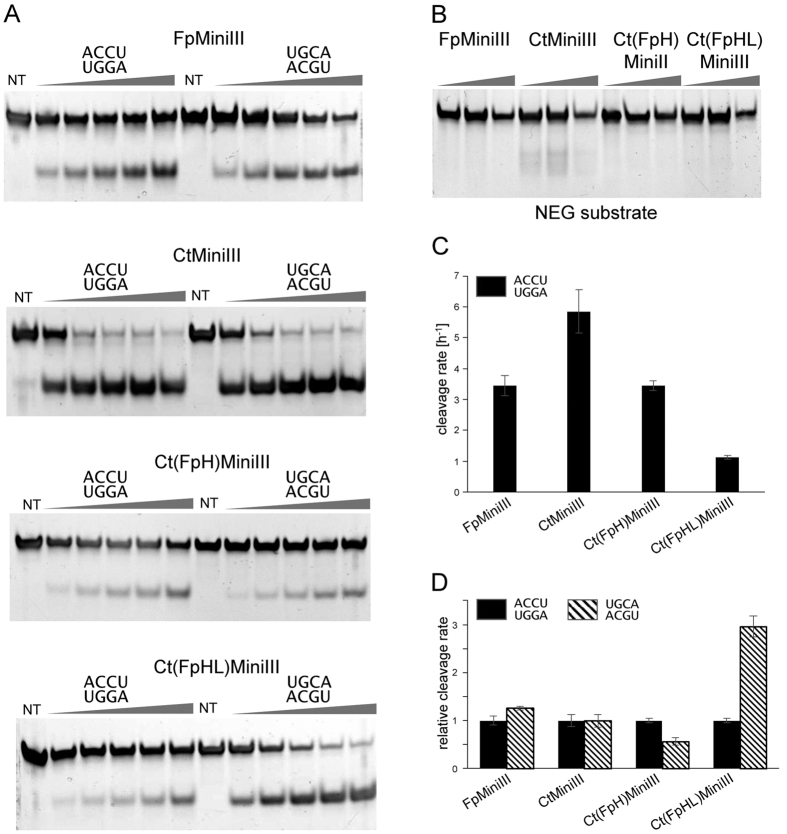
Sequence preference of swap variants in multiple turnover assay. Cleavage assays were performed with 2.5 μM substrates in enzyme:substrate ratio 1:10 for CtMiniIII and Ct(FpHL)MiniIII and 1:20 for FpMiniIII and Ct(FpH)MiniIII. (**A**) Comparison of cleavage activity on POS-ACCU and POS-UGCA substrates. Aliquots were collected after 0.5, 1, 1.5, 2 and 2.5 hours of reaction. (**B**) Cleavage of NEG substrate without preferred sequence. Aliquots were collected after 1, 2 and 3 hours of cleavage. Some unspecific cleavage of NEG substrate can be observed but only in CtMiniIII reaction transient accumulation of cleavage products is visible. (**C**) Comparison of the POS-ACCU cleavage rates. (**D**) Sequence preference of the swap variants and native enzymes. Cleavage rate of POS-UGCA substrate for each enzyme is normalized to the POS-ACCU cleavage rate of the same enzyme.

Selected swapped variants of CtMiniIII were assayed in multiple turnover cleavage reaction on two substrates POS-ACCU and POS-UGCA (Fig. 5) that contained the same target sequence as 910-ACCU and 910-UGCA molecules, respectively, which were used as substrates in single turnover assays. In contrast to the single turnover assay on longer dsRNA (Fig. 4), no increase in enzymatic activity was observed for Ct(FpH)MiniIII and Ct(FpHL)MiniIII, compared to native enzymes. On the contrary, Ct(FpHL)MiniIII activity was decreased at least threefold in comparison to FpMiniIII (Fig. 5C). A threefold preference for POS-UGCA over POS-UCCA was observed for the Ct(FpHL)MiniIII variant (Fig. 5D), which is in agreement with the single turnover assay results. On the other hand no preference for POS-UGCA substrate was observed for FpMiniIII.

### Cleavage sequence selection is not strictly dependent upon preferential binding

In a previous report we showed that deletion of the α5b-α6 loop in BsMiniIII did not alter the binding of this enzyme to dsRNA with a preferred cleavage sequence^17^. We hypothesized that the α5b-α6 loop is involved in selecting the cleavage site and further posited that this selection occurred at the step of cleavage itself and did not involve selective binding. In the current study we measured the dissociation constants of several Mini-III RNases and CtMiniIII swap variants for two 28 bp dsRNAs, one that contained the preferred cleavage site from the 910 S substrate (POS-ACCU) and the other with the same nucleotide composition but derived from the other fragment of 910 S sequence, in which no cleavage could be detected (NEG). No clear preference for binding the cleavage site was found (Table 2, Supplementary Fig. S10). The only exception was BsMiniIII, but even in this case, the difference in affinity was only twofold and the affinity for the preferred cleavage site was lower than for the uncleaved dsRNA. All of the measured K_D_ values were within the range of 70 nM to 2.25 μM, with the exception of FpMiniIII, in which no binding saturation was observed at the range of concentrations tested, indicating that the K_D_ of this enzyme was substantially higher. These results suggest that Mini-III enzymes do not select their cleavage sequence at the substrate binding step, but most probably they bind dsRNA irrespective of its sequence. In other words, as there is no difference in binding cleavable vs not-cleavable substrates, the binding preference is unlikely to be important for the cleavage site selection.

## Discussion

The presented results led to four important findings:

- Preference for cleaving distinct sequences in dsRNA is a common feature of Mini-III RNases
- There is a clear diversity of target sequences preferred by MiniIII enzymes studied in this work although they can all be summarized by the WSSW consensus sequence
- Helix α4 and loop α5b-α6 are involved in sequence selection
- Sequence preference for dsRNA cleavage is not a result of preferential substrate binding

In our previous study we showed that Mini-III RNase from *B. subtilis* is able to cleave long dsRNA in a sequence-dependent manner^17^. In the present study, we report that this is a common feature of the Mini-III subfamily of RNase III enzymes (class 4). We also found that swapping two structural elements, the α4 helix and α5b-α6 loop, between different MiniIII enzymes, led to significant changes in the catalytic activity and in some cases to alterations in sequence preference of the chimeric enzymes. These results suggest the involvement of both structural elements in catalytic activity and sequence selection.

Sequence specificity of the canonical RNase III from *E. coli*, has been only partially characterized; for instance there are no data concerning limited cleavage of long dsRNA molecules. In our own experiments we were unable to obtain discernible bands in limited cleavage of Φ6 dsRNA with RNase III (Supplementary Fig. S1). Most information has been obtained from *in vitro* studies with the μR1.1 RNA and its derivatives, composed of a relatively short RNA chain that forms a double stranded stem of 13–25 base pairs, including wobble G-U base pairs^18^,^19^. In these RNAs the cleavage site is located in a double-stranded region at the very end of the stem. These cleavage events are determined by recognition of two regions of the stem: (*i*) the distal box (UU/AG) at positions 10–11 from the center of the cleavage, and (*ii*) the proximal box (consensus sequence CWUW/WAWG) at positions 2–5 from the center of the cleavage. No sequence preference was observed for the central dinucleotide at the RNase III cleavage site.

The residues in the consensus of the preferred cleavage sequence of Mini-III enzymes, which were determined in this work based on limited cleavage of Φ6 dsRNA, and residues in the consensus sequence of *E. coli* RNase III have different localization in relation to the cleavage site. For Mini-III enzymes no conservation is observed for positions corresponding to the proximal box and the distal box. The most conserved region of four base pairs directly encompasses the cleavage site. Some additional preferences are exhibited by several of the enzymes at the distance of 4–5 residues from the cleavage site. The differences in target selection between class 1 (canonical) and class 4 (Mini-III) RNase III enzymes may be explained based on different structures of the two classes of the enzymes and differences in the enzyme-substrate contacts.

The first prominent difference in substrate recognition between the two classes of enzymes is the fact that RNase III recognizes the distal box, whereas the high throughput data presented in this work indicate that Mini-III RNases do not show preferences toward any sequence located at a distance longer than 5 bp from the cleavage site. For *E. coli* RNase III the distal box forms contacts with RBM4, which is located in a long loop between helices α5 and α6 12. This region in Mini-III RNases is replaced by a short helix α5b followed by a loop α5b-α6, which have been shown to be indispensable for the cleavage of the preferred sequence in dsRNA substrate^17^. We speculate that due to the structural differences between class 1 and class 4 enzymes, this region in Mini-III RNase is involved in interactions with the part of the substrate encompassing the cleavage site and not with the distal box.

The second prominent difference between the two classes of RNases III is the recognition by canonical RNase III of the so-called proximal box, formed by nucleotides 2–5 from the center of the cleavage. Nucleotides 5–6 form contacts with RBM1, an element located in dsRBD, which is absent from all Mini-III enzymes^11^,^12^,^19^. A common element between class 1 and 4 RNases III is helix α4, which in canonical RNase III enzymes forms the RBM3 element that interacts with the second and third nucleotide from the center of the cleavage site. We have shown that swapping of helices α4 between Mini-III enzymes resulted in an alteration of sequence preferences and cleavage activities. Therefore, we hypothesize that this helix plays a similar role in both class 1 and class 4 RNase III enzymes, and interacts with nucleotides in close proximity of the cleavage site. Since the sequence preference observed for Mini-III enzymes is mostly limited to residues 1 and 2 from the center of the cleavage site, it is possible that the interaction of helix α4 in this family is shifted one nucleotide towards the center of the target site relative to the canonical RNase III. It is worth noting that amino acid sequences in two Mini-III regions implicated in sequence preference, helix α4 and loop α5b-α6, are rather divergent, which might explain the observed differences in sequence preferences of the studied Mini-III RNases.

All the above analyses of the structural basis of sequence recognition by Mini-III enzymes would benefit greatly from the availability of a high-resolution structure for the nuclease-RNA complex. So far, our numerous attempts to crystalize Mini-III enzymes in a complex with dsRNA have unfortunately been unsuccessful.

An important element that influences the substrate preference and mechanism of action of RNases III is their domain composition. The majority of known RNA-binding proteins have modular structures and include one or more specialized RNA-binding domains^20^. Alone, these domains often bind short RNA stretches with weak affinity, however by existing in multiple copies, they endow a protein with the ability to bind RNA with high specificity and affinity. Thereby, RNA-binding modules of the same or of different structural types combined with enzymatic domains define the targets of many enzymes acting on RNA. Most members of the RNase III family (including canonical RNase III enzymes, Dicer and Drosha) contain dsRBD and this domain is responsible for dsRNA substrate binding by these enzymes. For example, for *E. coli* RNase III it was demonstrated that dsRBD deletion severely impairs this enzyme for RNA cleavage under standard *in vitro* conditions^21^, suggesting that interactions of dsRBD with RNA are crucial to form a cleavage-competent complex of RNase III with its substrate^22^. Among RNases III, the unique property of Mini-III enzymes is that they are single-domain proteins containing only the RNase III catalytic domain. The role of additional domains can be played by interacting proteins. For example studies carried out to date by the Condon group indicate that efficient cleavage of pre-rRNA can be achieved by Mini-III only in the presence of the L3 protein, which enables high affinity binding of the enzyme to the substrate RNA and thereby might serve as a functional analog of an RNA binding domain, e.g. dsRBD in *E. coli* RNase III^15^. We were unable to see any effect of L3 protein on target selection in cleavage of a long dsRNA of Φ6 bacteriophage (Supplementary Fig. S2).

Majority of cleavage assays used in this study to investigate Mini-III sequence preferences were performed in single turnover conditions. We have repeated selected assays in multiple turnover settings on smaller dsRNA substrates. The results of these assays on swapped variants of Mini-III do not show as many differences between these enzymes as were apparent in single turnover conditions, such as substantial increase of the catalytic activity in variant Ct(FpH) or differences in cleavage of 910-ACCU and 910-UGCA substrates by CtMiniIII and FpMiniIII. There are several factors that can explain these differences. For practical reasons, we used substrate concentration of 2.5 μM, which is not substantially higher than the measured dissociation constants (Table 2). In such conditions most probably both product release and rebinding of a new substrate steps had big impact on measured cleavage rates, which may obscure differences at the cleavage step that in turn are emphasized in a single turnover assay. Such effect is most noticeable for FpMiniIII, for which K_D_ is significantly higher than for other MiniIII enzymes. This can explain the lack of preference of this enzyme for the UGCA sequence in the multiple turnover assay, which was observed under single turnover conditions. Based on the binding affinity measurements we postulate that substrate selection takes place at the cleavage step, as no preferential binding of the “cleavable” substrate over “uncleavable” one was observed (Table 2). Therefore, in this case the single turnover assay is more informative to study the sequence selection phenomenon. It is worth emphasizing that for a similar reason, the single turnover assay is commonly used in investigations concerning sequence specificity and its alteration, for different enzymes acting on a double-stranded DNA^23^,^24^,^25^. Besides, the endonucleolytic activity of Mini-III enzymes is rather low. Therefore, the measurement of multiple turnover cleavage requires long incubation times, at which enzyme and substrate stability becomes an issue.

In our experiments we observed small differences between high-throughput sequencing data and the results of *in vitro* cleavage assays of isolated dsRNA substrates. This indicates that possibly factors other than just sequence consensus recognition are involved in the selection of cleavage sites by Mini-III enzymes. One such factor could be altered geometry of RNA in the cleaved sequence or in its close proximity, perhaps influenced by the surrounding sequence. We analyzed target sequences for evident and recurrent sequence-dependent distortions in base-step geometries using values for shift, slide, rise, tilt, roll, and twist from the RNA STEPS database^26^, but we did not find such anomalies (data not shown). This makes the involvement of altered geometry in sequence preference mechanism unlikely.

The molecular basis of the target sequence selection by Mini-III enzymes is not known but RNA binding assays indicate that preferential binding is not involved in this process. An alternative possibility is the contribution of a mechanism described for *E. coli* RNase III, based on a combination of biochemical and structural data on the enzyme-RNA complex of its close homolog. In this mechanism antidetereminants and positive determinants within the RNA sequence mask or expose particular phosphodiester bonds for the endoRNase cleavage by local alteration of the dsRNA structure^12^,^18^. A similar mechanism may explain the sequence preference of Mini-III enzymes. Another possible mechanism are conformational changes of the dsRNA substrate upon Mini-III binding required to generate a cleavage-proficient complex. Such a conformational change may be more likely to occur for some of the target sequences than for others. Such a behavior was observed for many restriction endonucleases and included various distortions of the substrate^27^,^28^. Although restriction enzymes do bind dsDNA in a sequence-independent manner, this binding is typically much weaker compared to the binding of the cognate sequence^29^,^30^,^31^. There are, however, restriction endonucleases, such as the Cfr9I, which bind cognate and non-cognate sequences with similar affinities^32^. One possibility is that a similar lack of selectivity at the substrate binding step is a reason why the sequence preference of Mini-III is rather weak. Importantly, the preference of BsMiniIII to cleave pre-RNA *in vivo* is brought about by the presence of ribosomal protein L3 in close proximity to the cleavage site, as well as the complicated secondary and tertiary structure of the whole substrate molecule^15^.

We demonstrate that in addition to targeting pre-rRNA *in vivo*, Mini-III RNases exhibit preferences for sequences in dsRNA, dictated by structural elements within the catalytic domain. We found that this sequence specificity is exerted not at the level of substrate binding, but at the level of cleavage. This is a unique feature of Mini-III enzymes, since all other RNase III superfamily members use the dsRBD domain(s), rather than the catalytic domain, to bind dsRNA substrates with high affinity^12^,^33^,^34^,^35^ see also ref. 36.

Identification of new structural elements in the RNase III catalytic domain that participate in target selection, together with the discovery of a diversity of solutions used by RNase III enzymes to achieve this goal, provides a platform for engineering substrate specificity in Mini-III enzymes to make them useful tools for RNA research.

## Methods

### Cloning, protein expression, and purification

Bacterial genomic DNA was isolated from reconstituted cultures that were obtained from the DSMZ strain collection (Germany; Table 1). DNA was purified by phenol extraction and ethanol precipitation. The Mini-III coding sequences and *B. subtilis* L3 coding sequence were amplified from genomic DNA by polymerase chain reaction (PCR) with Pfu DNA polymerase and the primers that are listed in Supplementary Table S4. The reaction products were digested with NdeI and XhoI and inserted into a pET28a vector that was cleaved with the same enzymes. The resulting plasmids encoded endoRNases with an N-terminal His_6_-tag. The L3 coding sequence was prepared in the same way but cloned into pET30a vector resulting in the plasmid encoding L3 protein with a C-terminal His_6_-tag.The E106Q substitution in BsMiniIII catalytic center, which leads to complete inactivation of BsMiniIII endonucleolytic activity, was introduced by the inside-out PCR amplification of wt BsMiniIII construct with E106Qfv and E106Qrev primers, phosphorylation of PCR product and recircularization with T4 DNA ligase. All the constructs were verified by DNA sequencing.

To construct plasmids that encoded chimeric versions of Mini-IIIs with parts of the α4 helix and α5b-α6 loop that were swapped, original plasmids that expressed one Mini-III (acceptor) were subjected to inside-out PCR with Pfu DNA polymerase that amplified the entire template, with the exception of the sequence that encoded the structural element that was to be swapped (Supplementary Table S5). The PCR products were treated with T4 polynucleotide kinase and ligated with short double-stranded oligonucleotides that contained a corresponding sequence from the other Mini-III (donor). Inserts that replaced the sequence encoding the α5b-α6 loop were created by filling in 5′-protruding sticky ends formed by annealing partially complementary oligonucleotides with the Klenow fragment of DNA polymerase I. Constructs with a confirmed correct orientation of the insert were selected after DNA sequencing. The nomenclature of the chimeras that were obtained indicates (in parentheses) the source and identity of the swapped element. For example, CtMiniIII with part of the α4 helix from FpMiniIII was named Ct(FpH)MiniIII. CtMiniIII with part of the α5b-α6 loop that was derived from FpMiniIII was named Ct(FpL)MiniIII (Fig. 4 A). The following chimeras were constructed: Ct(FpH)MiniIII, Ct(FpL)MiniIII, Fp(CtH)MiniIII, Fp(CtL)MiniIII, Bs(FpH)MiniIII, Bs(FpL)MiniII, Sp(FpH)MiniIII, Sp(FpL)MiniIII, and Ct(FpHL)MiniIII, in which both structural elements of CtMiniIII were replaced with the corresponding sequences of FpMiniIII.

To obtain recombinant proteins, plasmids were transformed into the *E. coli* BL21 (DE3) strain. Enzymes were produced by 24-h autoinduction at 37 °C 37. Cells were harvested by centrifugation, resuspended in buffer L0 (50 mM Tris HCl [pH 7.5] and 300 mM NaCl) and lysed by a single passage through a Constant Systems cell disruptor at 20 kpsi. Lysates were clarified by centrifugation at 50,000 × *g* for 20 min at 4 °C. The proteins were purified by Ni-NTA affinity chromatography on a His-Select Nickel Affinity Gel (Sigma). After 1-h incubation at 4 °C with the clarified lysate, the resin was washed with 10 bed volumes of wash buffer L0, applied to the disposable gravity flow column, washed with 30 volumes of wash buffer 1 (buffer L0 + 10 mM imidazole, pH 7.5), 10 bed volumes of wash buffer 2 (50 mM Tris HCl [pH 7.5], 2 M NaCl, and 10 mM imidazole), and 10 bed volumes of wash buffer 3 (buffer L0 with 20 mM imidazole). Purified recombinant proteins were then eluted with elution buffer (buffer L0 with 250 mM imidazole). Fractions that corresponded to the second and third column volumes were collected, pooled, and supplemented with glycerol to a final concentration of 50%. Samples were stored at −20 °C.

Protein concentrations were calculated based on absorption at 280 nm, measured with a NanoDrop 1000 spectrophotometer. Homogeneity of the protein samples, assessed by SDS-PAGE, was higher than 85% (Supplementary Fig. S11).

### RNA substrate preparation

Φ6 phage was produced and purified as described previously^38^ with minor modifications. LB broth was used as a liquid medium. Phage particles were concentrated by PEG precipitation and purified by equilibrium CsCl gradient ultracentrifugation at 150,000 × *g* for 24 h at 4 °C. Purified virions were diluted five-fold in 10 mM potassium phosphate (pH 7.1), 1 mM MgSO_4_, pelleted by centrifugation at 70,000 × *g* for 75 min at 4 °C, and resuspended in TE buffer (10 mM TrisHCl [pH 7.5, 1 mM ethylenediaminetetraacetic acid [EDTA]). dsRNA was isolated by phenol extraction and ethanol precipitation. Purified dsRNA was dissolved in 1 mM sodium citrate (pH 6.4) at 1 mg/ml, aliquoted, and stored at −20 °C.

Isolated fragments of the Φ6 genome were prepared by enzymatic *in vitro* synthesis. The product of the reverse transcription of Φ6 dsRNA with random hexamer primers and Maxima Reverse Transcriptase (Thermo Scientific) was used as the PCR template to produce dsDNA that was equivalent to the desired phage genome flanked by the T7 RNA polymerase promoter (TAATACGACTCACTATAGGG) on one end and the Φ6 RNA polymerase promoter (GGAAAAAAA) on the other end (Supplementary Table S6). These PCR products were used to produce defined dsRNA substrates with a replicator RNAi Kit (Thermo Scientific).

To generate a template for dsRNA synthesis of the 910 S RNA molecule and its variants that had various substitutions, the dsDNA template used to produce dsRNA was cloned into the SmaI site of the pUC19 plasmid, thus providing the pUC910S plasmid. Substitutions of each position in the cleavage site were obtained by inside-out PCR amplification of the pUC910S plasmid with three sets of the primers that contained a degenerate sequence in the cleavage site (Supplementary Table S7). The 5′ ends of the PCR products were phosphorylated with T4 polynucleotide kinase and circularized with T4 DNA ligase. The resulting plasmids were subjected to DNA sequencing to characterize substitutions in each clone.

The 28 bp dsRNAs that were used for the filter binding assays and for the multiple turnover cleavage assays were generated by annealing complementary RNA oligonucleotides (Supplementary Table S6). For binding assays dsRNAs were labeled with [γ−33P]ATP using a T4 polynucleotide kinase. Labeled dsRNA molecules were purified using mini Quick Spin Oligo Columns (Roche).

### dsRNA cleavage assays

The cleavage buffers and reaction temperatures that were used for each enzyme are listed in Table 1. The enzyme and substrate concentrations and reaction times in each experiment are described in the Results section. The reactions were terminated by mixing the reaction aliquot with the gel loading buffer that contained EDTA (10 mM final concentration) and a 1/10 volume of a 1:1 phenol:chloroform mixture. The products were separated by non-denaturing gel electrophoresis in 1.5% agarose (Φ6 bacteriophage genome) and in 8% or 15% polyacrylamide for the single turnover and multiple turnover cleavage assays respectively, stained with ethidium bromide, and visualized with ultraviolet light using the LAS 4010 imaging system (GE Healthcare). Cleavage kinetics were measured based on a densitometric analysis of each reaction time point on the electropherograms using ImageQuantTL software (GE Healthcare). Cleavage rates were calculated from the initial linear part of the reaction progress curves. Measurements were made in triplicate.

### RNA sequencing analyses

A 5 μg sample of the Φ6 genome was used as a substrate in a time-limited cleavage reaction, to achieve a 10% decrease in the intensity of the substrate bands. The cleavage reaction of the substrate was performed for 10 min for the majority of the enzymes, with the exception of SeMiniIII and TmMiniIII, in which the reaction times were 40 and 5 min, respectively. The preparation of the sequencing libraries was performed as described previously^17^. High-throughput sequencing was performed using a MiSeq (Illumina) platform at Genomed (Warsaw, Poland).

The processing of sequencing data was performed as described previously^17^. Two hundred of the most frequent cleavage sites in each experiment were selected for further analysis. Fourteen-nucleotide-long sequences of these sites were used to build consensus sequences of the preferred cleavage sites for each enzyme using MEME software^39^ and default parameters, with the exception of setting the minimum width of the motifs to 14.

### Filter binding assay

Nitrocelullose filters (GE Healthcare) were soaked for 30 min at room temperature in binding buffers that were identical to the cleavage buffers, with the exception that MgCl_2_ was replaced with 1 mM CaCl_2_. The binding reactions were performed for 1 h at room temperature in a total volume of 50 μl with 0.0015 μM ^33^P end-labeled 28 bp dsRNA and 0.03-10 μM of enzyme. The binding reactions were filtered through nitrocellulose equilibrated with binding buffer, followed by three 150-μl washes with the binding buffer. The filters were dried, and radioactivity was recorded autoradiographically using the Storage Phosphor Screen (GE Healthcare) and scanned with Typhoon Trio+ Imager (GE Healthcare). The quantitative analysis of the autoradiograms was performed using ImageQuant TL software (GE Healthcare). Dissociation constants were calculated by GraphPad Prism 6 (GraphPad Software, Inc.) with the single-site-specific binding model.

### Bioinformatics analysis

Sequences of 23 S rRNA genes were obtained from NCBI database along with 100-nucleotide long flanking regions and aligned using T-Coffee web server^40^ with standard server parameters. 14–16 nucleotide long sequences flanking in the alignment the position of the known 5′ and 3′ BsMiniIII cleavage sites in *B. subtilis* 23 S pre-rRNA were extracted and used in secondary structure prediction with CentroidFold web server^41^ using default parameters. Secondary structure prediction was carried out using PSIPRED using default parameters^42^.

## Additional Information

**How to cite this article**: Głów, D. *et al*. Identification of protein structural elements responsible for the diversity of sequence preferences among Mini-III RNases. *Sci. Rep.* **6**, 38612; doi: 10.1038/srep38612 (2016).

**Publisher's note:** Springer Nature remains neutral with regard to jurisdictional claims in published maps and institutional affiliations.

## Supplementary Material

Supplementary Materials

## Figures and Tables

**Table 1:** Mini-III RNases that were used in the present study.

Enzyme	Microorganism	Strain	Gene identifier (GI)	DSMZ no.	Reaction temperature (°C)	Cleavage buffer^1^
BsMiniIII	*Bacillus subtilis*	168	2632362	402	37	Bs
CkMiniIII	*Caldicellulosiruptor kristjanssonii*	I77R1B	311792827	12137	65	G1
CrMiniIII	*Clostridium ramosum*	113-I	167756029	1402	37	Bs
CtMiniIII	*Clostridium thermocellum*		125974551	1237	55	B1
FnMiniIII	*Fusobacterium nucleatum subsp. nucleatum*	1612 A	19704899	15643	37	BG
FpMiniIII	*Faecalibacterium prausnitzii*	A2-165	160943938	17677	37	Bs
SeMiniIII	*Staphylococcus epidermidis*	PCI 1200	27467211	1798	37	R
TmMiniIII	*Thermotoga maritima*	MSB8	15644486	3109	65	R
TtMiniIII	*Thermoanaerobacter tengcongensis (Caldanaerobacter subterraneus subsp. tengcongensis)*	MB4	20808680	15242	65	B1

^1^Each enzyme was tested in all buffers listed in Supplementary Table S1 to select conditions leading to well pronounced cleavage pattern.

**Table 2:** Dissociation constants for the preferred cleavage substrate and uncleaved dsRNA.

Protein	K_D (POS-ACCU)_	K_D (NEG)_	Protein concentration
BsMiniIII	0.14 μM (+/−0.02)^1^	0.07 μM (+/−0.01)	0.03125–1 μM
CtMiniIII	1.14 μM (+/−0.11)	1.34 μM (+/−0.28)	0.3125–10 μM
FpMiniIII	> 100 μM	19.9 μM (+/−3.20)	0.3125–10 μM
SeMiniIII	0.16 μM (+/−0.02)	0.19 μM (+/−0.02)	0.0375–1.2 μM
Ct(FpH)MiniIII	2.62 μM (+/−0.38)	1.94 μM (+/−0.23)	0.14–4.6 μM
Ct(FpL)MiniIII	1.33 μM (+/−0.33)	1.8 μM (+/−0.28)	0.156–5 μM
Ct(FpHL)MiniIII	2.17 μM (+/−0.59)	2.25 μM (+/−0.86)	0.109–3.5 μM

The equilibrium dissociation constants (K_D_) were obtained from global fitting of the results of triplicate measurement in nitrocellulose filter binding assay (see Materials and Methods) at enzyme concentration ranges listed in the right column to the one site specific binding model (with the Prism 6.0 software) ^1^. Brackets indicate the standard error of fitting.

## References

[b1] Czaja R. . RNase T1 variant RV cleaves single-stranded RNA after purines due to specific recognition by the Asn46 side chain amide. Biochemistry 43, 2854–2862 (2004). 15005620 10.1021/bi035961f

[b2] Saida F., Uzan M. & Bontems F. The phage T4 restriction endoribonuclease RegB: a cyclizing enzyme that requires two histidines to be fully active. Nucleic Acids Res. 31, 2751–2758 (2003). 12771201 10.1093/nar/gkg377 PMC156712

[b3] Lebars I., Hu R. M., Lallemand J. Y., Uzan M. & Bontems F. Role of the substrate conformation and of the S1 protein in the cleavage efficiency of the T4 endoribonuclease RegB. J. Biol. Chem. 276, 13264–13272 (2001). 11118457 10.1074/jbc.M010680200

[b4] Endo Y., Gluck A., Chan Y. L., Tsurugi K. & Wool I. G. RNA-protein interaction. An analysis with RNA oligonucleotides of the recognition by alpha-sarcin of a ribosomal domain critical for function. J. Biol. Chem. 265, 2216–2222 (1990). 2298746

[b5] Dow-Tien C., Yuan-Jhih T. & Alan L. Creating a ribonuclease T-tat that preferentially recognizes and hydrolyzes HIV-1 TAR RNA *in vitro* and *in vivo*. Nucleic Acids Res. 36, 963–969 (2008). 18086702 10.1093/nar/gkm1118 PMC2241915

[b6] Choudhury R., Tsai Y. S., Dominguez D., Wang Y. & Wang Z. Engineering RNA endonucleases with customized sequence specificities. Nat. Commun. 3, 1147 (2012). 23093184 10.1038/ncomms2154 PMC3612931

[b7] Usman N., Beigelman L. & McSwiggen J. A. Hammerhead ribozyme engineering. Curr. Op. Struct. Biol. 6, 527–533 (1996). 10.1016/s0959-440x(96)80119-9 8794164

[b8] Feldman A. R. & Sen D. A new and efficient DNA enzyme for the sequence-specific cleavage of RNA. J. Mol. Biol. 313, 283–294 (2001). 11800557 10.1006/jmbi.2001.5058

[b9] Murtola M., Wenska M. & Stromberg R. PNAzymes That Are Artificial RNA Restriction Enzymes. J. Am. Chem. Soc. 132, 8984–8990 (2010). 20545354 10.1021/ja1008739

[b10] Price A. A., Sampson T. R., Ratner H. K., Grakoui A. & Weiss D. S. Cas9-mediated targeting of viral RNA in eukaryotic cells. Proc. Natl. Acad. Sci. USA 112, 6164–6169 (2015). 25918406 10.1073/pnas.1422340112 PMC4434742

[b11] Redko Y., Bechhofer D. H. & Condon C. Mini-III, an unusual member of the RNase III family of enzymes, catalyses 23S ribosomal RNA maturation in B. subtilis. Mol. Microbiol. 68, 1096–1106 (2008). 18363798 10.1111/j.1365-2958.2008.06207.x

[b12] Gan J. H. . Structural insight into the mechanism of double-stranded RNA processing by ribonuclease III. Cell 124, 355–366 (2006). 16439209 10.1016/j.cell.2005.11.034

[b13] Kim K., Sim S. H., Jeon C. O., Lee Y. & Lee K. Base substitutions at scissile bond sites are sufficient to alter RNA-binding and cleavage activity of RNase III. FEMS Microbiol. Lett. 315, 30–37 (2011). 21133991 10.1111/j.1574-6968.2010.02169.x

[b14] Krinke L. & Wulff D. L. The cleavage specificity of RNase III. Nucleic Acids Res. 18, 4809–4815 (1990). 1697676 10.1093/nar/18.16.4809 PMC331951

[b15] Redko Y. & Condon C. Ribosomal protein L3 bound to 23S precursor rRNA stimulates its maturation by Mini-III ribonuclease. Mol. Microbiol. 71, 1145–1154 (2009). 19154332 10.1111/j.1365-2958.2008.06591.x

[b16] Hotto A. M. . Arabidopsis Chloroplast Mini-Ribonuclease III Participates in rRNA Maturation and Intron Recycling. Plant Cell 27, 724–740 (2015). 25724636 10.1105/tpc.114.134452 PMC4558656

[b17] Glow D. . Sequence-specific cleavage of dsRNA by Mini-III RNase. Nucleic Acids Res. 43, 2864–2873 (2015). 25634891 10.1093/nar/gkv009 PMC4357697

[b18] Zhang K. & Nicholson A. W. Regulation of ribonuclease III processing by double-helical sequence antideterminants. Proc. Natl. Acad. Sci. USA 94, 13437–13441 (1997). 9391043 10.1073/pnas.94.25.13437 PMC28323

[b19] Pertzev A. V. & Nicholson A. W. Characterization of RNA sequence determinants and antideterminants of processing reactivity for a minimal substrate of Escherichia coli ribonuclease III. Nucleic Acids Res. 34, 3708–3721 (2006). 16896014 10.1093/nar/gkl459 PMC1540722

[b20] Lunde B. M., Moore C. & Varani G. RNA-binding proteins: modular design for efficient function. Nat. Rev. Mol. Cell Biol. 8, 479–490 (2007). 17473849 10.1038/nrm2178 PMC5507177

[b21] Sun W., Jun E. & Nicholson A. W. Intrinsic double-stranded-RNA processing activity of Escherichia coli ribonuclease III lacking the dsRNA-binding domain. Biochemistry 40, 14976–14984 (2001). 11732918 10.1021/bi011570u

[b22] Nicholson A. W. Ribonuclease III mechanisms of double-stranded RNA cleavage. Wiley Interdiscip. Rev. RNA 5, 31–48 (2014). 24124076 10.1002/wrna.1195 PMC3867540

[b23] Wenz C., Hahn M. & Pingoud A. Engineering of variants of the restriction endonuclease EcoRV that depend in their cleavage activity on the flexibility of sequences flanking the recognition site. Biochemistry 37 (1998). 10.1021/bi9719197 9485369

[b24] Parry D., Moon S. A., Liu H. H., Heslop P. & Connolly B. A. DNA Recognition by the EcoRV Restriction Endonuclease Probed using Base Analogues. J. Mol. Biol. 331, 1005–1016 (2003). 12927537 10.1016/s0022-2836(03)00861-1

[b25] Chahar S., Elsawy H., Ragozin S. & Jeltsch A. Changing the DNA recognition specificity of the EcoDam DNA-(adenine-N6)-methyltransferase by directed evolution. J. Mol. Biol. 395, 79–88 (2010). 19766657 10.1016/j.jmb.2009.09.027

[b26] Xin Y. & Olson W. K. BPS: a database of RNA base-pair structures. Nucleic Acids Res. 37, D83–88 (2009). 18845572 10.1093/nar/gkn676 PMC2686499

[b27] Winkler F. K. . The crystal structure of EcoRV endonuclease and of its complexes with cognate and non-cognate DNA fragments. EMBO J. 12, 1781–1795 (1993). 8491171 10.2210/pdb4rve/pdb PMC413397

[b28] Viadiu H. & Aggarwal A. K. Structure of BamHI bound to nonspecific DNA: a model for DNA sliding. Mol. Cell 5, 889–895 (2000). 10882125 10.1016/s1097-2765(00)80329-9

[b29] Langowski J., Pingoud A., Goppelt M. & Maass G. Inhibition of EcoRI action by polynucleotides - a characterization of the nonspecific-binding of the enzyme to DNA. Nucleic Acids Res. 8, 4727–4736 (1980). 6255431 10.1093/nar/8.20.4727 PMC324382

[b30] Alves J., Selent U. & Wolfes H. Accuracy of the EcoRV restriction endonuclease - binding and cleavage studies with oligodeoxynucleotide substrates containing degenerate recognition sequences. Biochemistry 34, 11191–11197 (1995). 7669777 10.1021/bi00035a026

[b31] Vipond I. B. & Halford S. E. Specific DNA recognition by EcoRV restriction endonuclease induced by calcium-ions. Biochemistry 34, 1113–1119 (1995). 7827059 10.1021/bi00004a002

[b32] Siksnys V. & Pleckaityte M. Catalytic and binding properties of restriction endonuclease Cfr9I. Eur. J. Biochem. 217, 411–419 (1993). 8223580 10.1111/j.1432-1033.1993.tb18260.x

[b33] Gan J. . Intermediate states of ribonuclease III in complex with double-stranded RNA. Structure 13, 1435–1442 (2005). 16216575 10.1016/j.str.2005.06.014

[b34] Wu H., Henras A., Chanfreau G. & Feigon J. Structural basis for recognition of the AGNN tetraloop RNA fold by the double-stranded RNA-binding domain of Rnt1p RNase III. Proc. Natl. Acad. Sci. USA 101, 8307–8312 (2004). 15150409 10.1073/pnas.0402627101 PMC420390

[b35] Wang Z., Hartman E., Roy K., Chanfreau G. & Feigon J. Structure of a yeast RNase III dsRBD complex with a noncanonical RNA substrate provides new insights into binding specificity of dsRBDs. Structure 19, 999–1010 (2011). 21742266 10.1016/j.str.2011.03.022 PMC3143303

[b36] Masliah G., Barraud P. & Allain F. H. RNA recognition by double-stranded RNA binding domains: a matter of shape and sequence. Cell Mol. Life Sci. 70, 1875–1895 (2013). 22918483 10.1007/s00018-012-1119-x PMC3724394

[b37] Studier F. W. Protein production by auto-induction in high-density shaking cultures. Protein Expres. Purifi. 41, 207–234 (2005). 10.1016/j.pep.2005.01.016 15915565

[b38] Vidaver A. K., Koski R. K. & Van Etten J. L. Bacteriophage phi6: a Lipid-Containing Virus of Pseudomonas phaseolicola. J. Virol. 11, 799–805 (1973). 16789137 10.1128/jvi.11.5.799-805.1973 PMC355178

[b39] Bailey T. L. . MEME SUITE: tools for motif discovery and searching. Nucleic Acids Res. 37, W202–W208 (2009). 19458158 10.1093/nar/gkp335 PMC2703892

[b40] Di Tommaso P. . T-Coffee: a web server for the multiple sequence alignment of protein and RNA sequences using structural information and homology extension. Nucleic Acids Res. 39, W13–17 (2011). 21558174 10.1093/nar/gkr245 PMC3125728

[b41] Sato K., Hamada M., Asai K. & Mituyama T. CENTROIDFOLD: a web server for RNA secondary structure prediction. Nucleic Acids Res. 37, W277–280 (2009). 19435882 10.1093/nar/gkp367 PMC2703931

[b42] McGuffin L. J., Bryson K. & Jones D. T. The PSIPRED protein structure prediction server. Bioinformatics 16, 404–405 (2000). 10869041 10.1093/bioinformatics/16.4.404

