# Supplementary material for: Identification of protein structural elements responsible for the diversity of sequence preferences among Mini-III RNases
Source: Sci Rep. 2016 Dec 7;6:38612. doi: 10.1038/srep38612 (PMC5141509; doi:10.1038/srep38612)
Supplement: Supplementary Materials [file srep38612-s1.pdf]

SUPPLEMENTARY MATERIALS

Identification of protein structural elements responsible for the diversity of sequence preferences among Mini-III RNases

Dawid Głów[1], Małgorzata Kurkowska[1], Justyna Czarnecka[1], Krzysztof Szczepaniak[1], Dariusz Pianka[1], Verena Kappert[1], Janusz M.Bujnicki[1], Krzysztof J. Skowronek[1,*]

[1]Laboratory of Bioinformatics and Protein Engineering, International Institute of Molecular and Cell Biology in Warsaw, ul. Ks. Trojdena 4, 02-109 Warsaw, Poland

* kskowronek@iimcb.gov.pl (Correspondence may also be addressed to Janusz M. Bujnicki: iamb@genesilico.pl.)

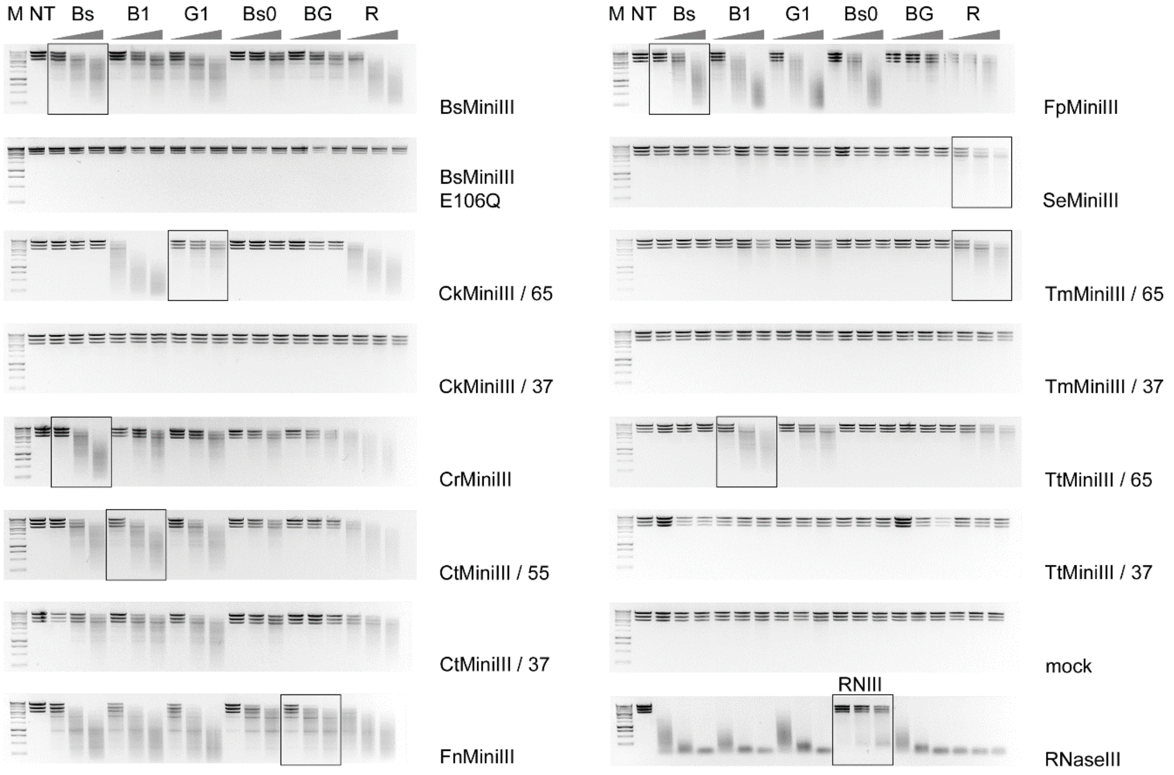

Figure S1. Cleavage activity of Mini-III enzymes in the set of buffers used in this study tested on Φ6 dsRNA (for buffers composition see Supplementary Table S1). All reactions were set up using 1.5 µg of dsRNA and the same amounts of enzymes as described in Figure 1. Aliquots were taken after 5, 10 and 10 min, with the exception of TtMiniIII, where aliquots were taken at 2, 4 and 6 min and SeMiniIII, where aliquots were taken at 20, 40 and 60 min. The buffer selected for each enzyme for the further assays is indicated by the frame. For thermophilic enzymes reactions were performed in two temperatures indicated after enzyme name. Results of treating the substrate RNA with BsMiniIII E106Q catalytic mutant, the mock purification from the strain carrying plasmid vector without an insert and 2 units of RNase III from *E. coli* (EURx) for 15, 30 and 45 min are shown as well. NT – an untreated Φ6 dsRNA. M – molecular weight marker (Perfect Plus 1kb DNA Ladder, EURx). RNIII – RNase III reaction buffer supplied by the vendor.

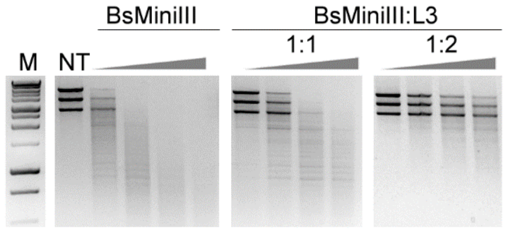

Figure S2. Effect of the *B. subtilis* L3 ribosomal protein on cleavage of Φ6 dsRNA by BsMiniIII. 2.5 µg of dsRNA was cleaved with 1.6 µg of BsMiniIII without L3 and in the presence of L3. BsMiniIII:L3 stoichiometry is indicated above the lines. Aliquots were taken after 15, 30, 45 and 60 minutes of reaction. M – molecular weight marker (Perfect Plus 1kb DNA Ladder, EURx); NT – untreated Φ6 dsRNA.

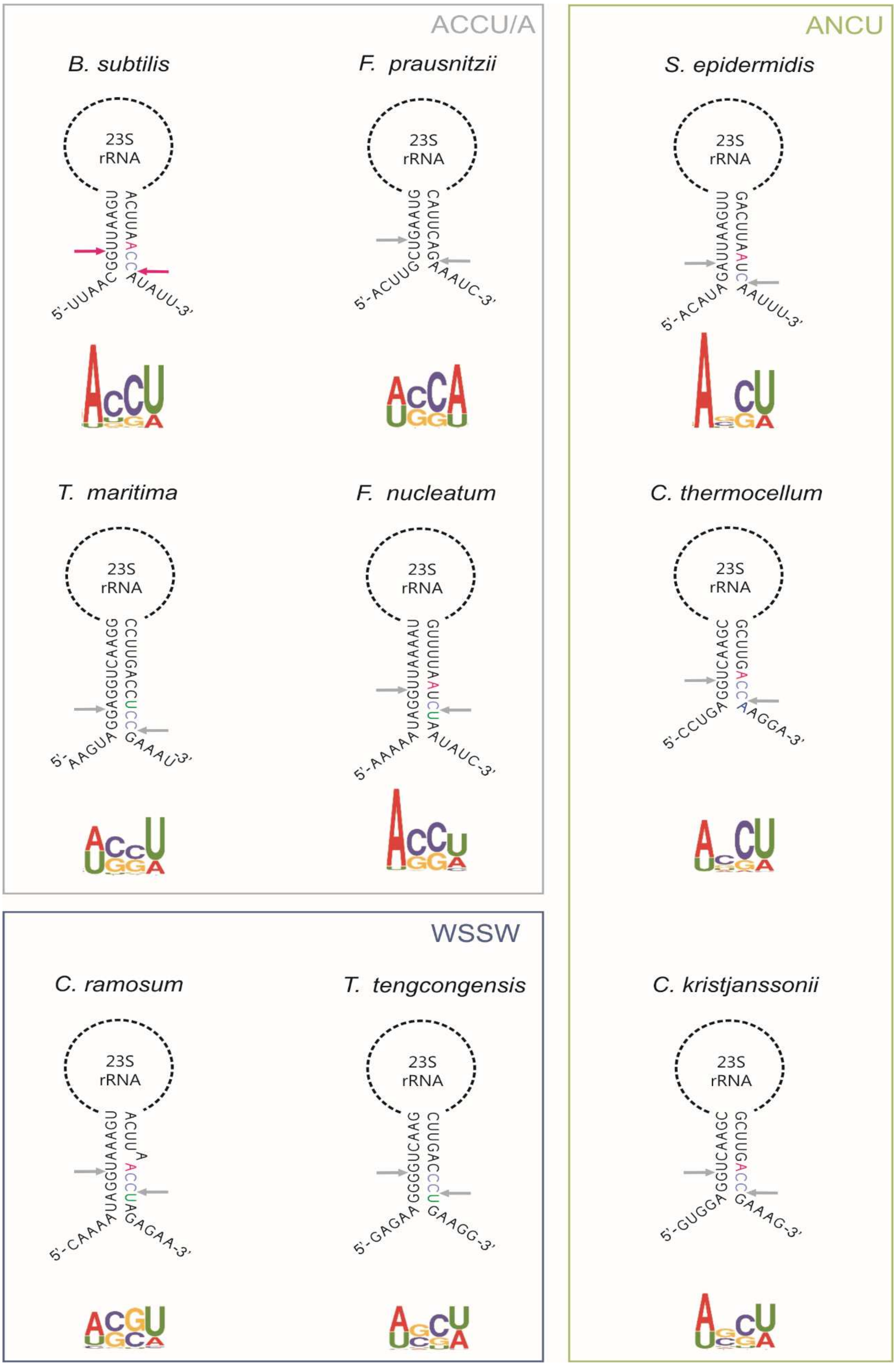

Figure S3. Predictions of the possible Mini-III substrates in 23S pre-rRNAs. The known BsMiniIII cleavage site is shown by the red arrows, hypothetical cleavage sites in the other organisms are show by the grey arrows.

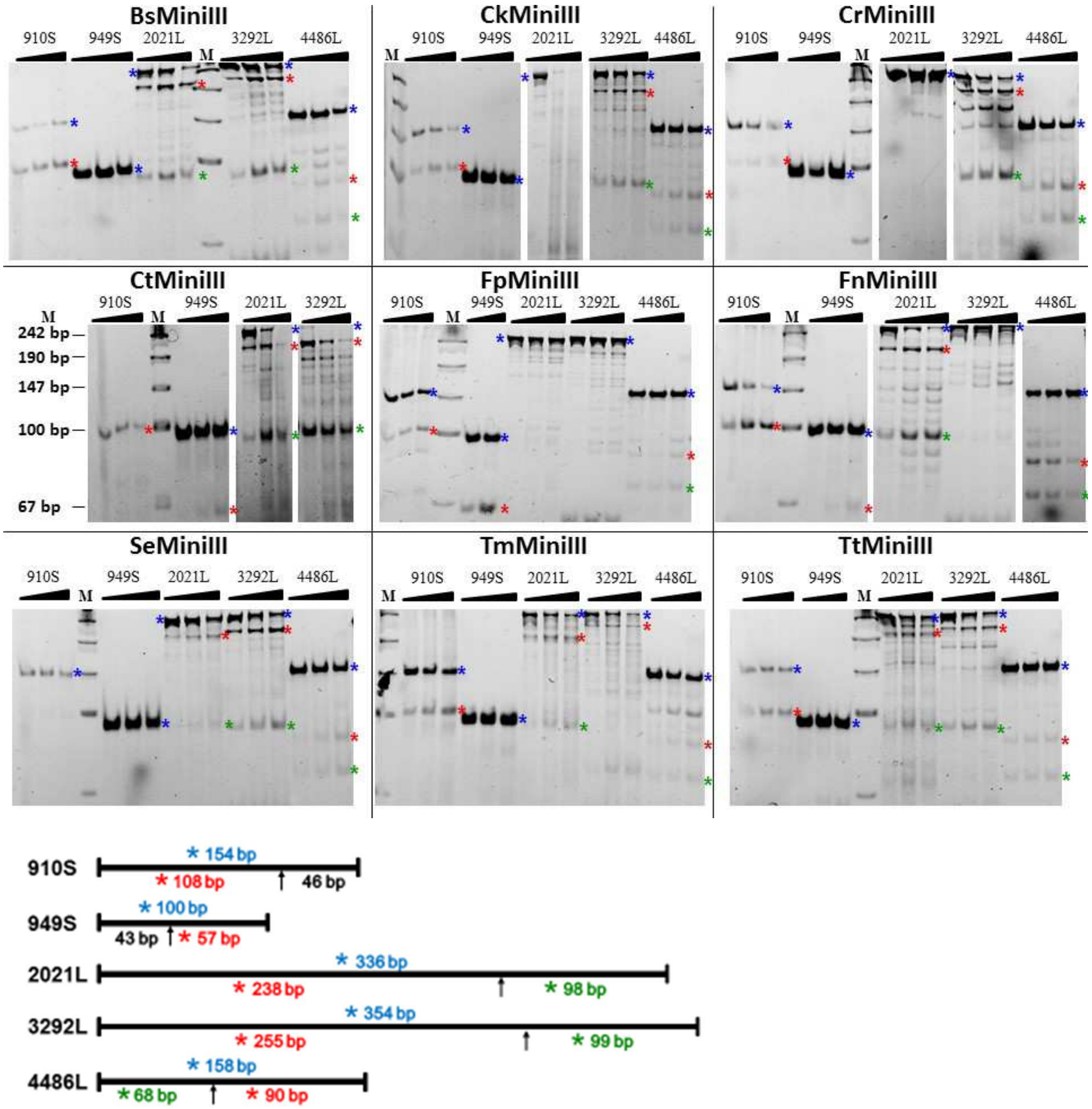

Figure S4. Cleavage assays of Mini-III enzymes on isolated fragments of the Φ6 genome. Substrate names are shown above the lanes (see Supplementary TableS2). dsRNA (0.3 µg) was digested with 3.3 µg BsMiniII, 80 ng CkMiniIII, 23.5 µg CrMiniIII, 5 µg CtMiniIII, 0.8 µg FnMiniIII, 1.1 µg FpMiniIII, 2.1 µg SeMiniIII, 0.185 µg TmMiniIII, and 11.5 ng TtMiniIII under favorable conditions for each enzyme. Aliquots were taken at 10, 20, and 30 min, with the exception of TtMiniIII and TmMiniIII, in which aliquots were taken at 5, 10, and 15 min, and SeMiniIII, in which aliquots were taken at 20, 40, and 60 min. M - dsDNA molecular weight marker. Schematic representation of the dsRNA substrates is shown on the lower panel. Lengths of the substrates are shown above the lines in blue; arrows indicate major cleavage sites and the lengths of the cleavage products are below the lines with fragments marked on the gels labeled red and green.

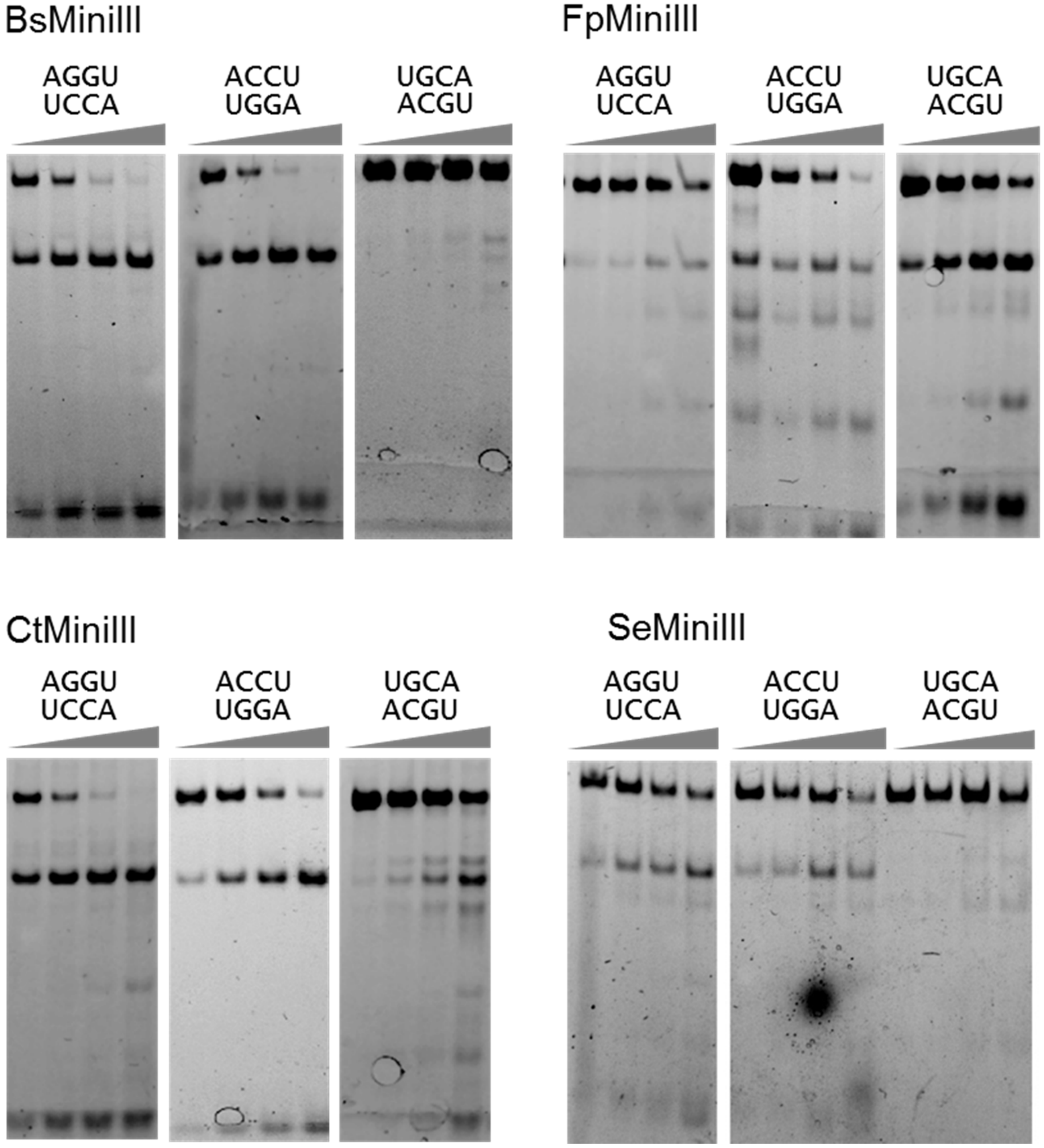

Figure S5. Cleavage of the 910S substrate sequence and selected substitution derivatives. dsRNA (0.4 µg) was digested with 3.47 µg of BsMiniII, 1.78 µg of CtMiniIII, 1.21 µg of FpMiniIII, and 48 µg of SeMiniIII under optimal conditions for each enzyme. Aliquots were taken at 15, 30, 60, and 120 min.

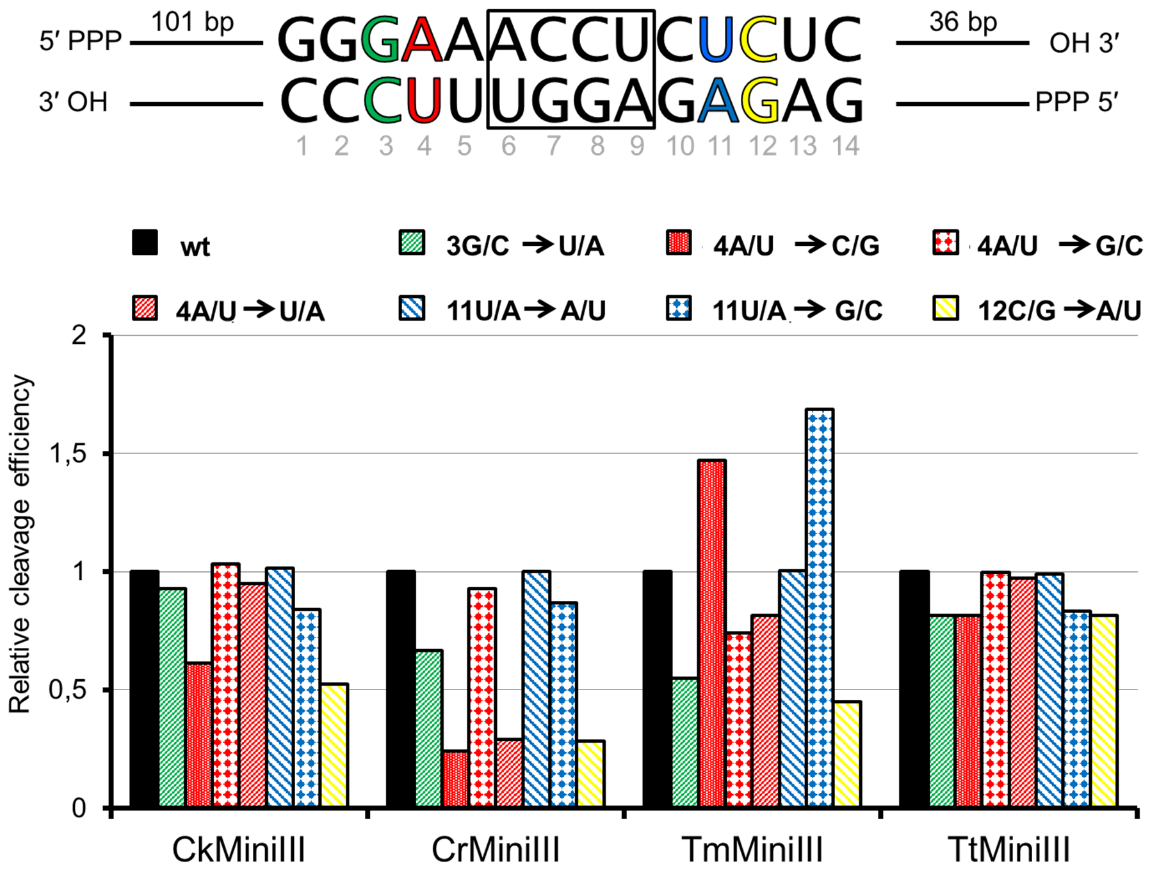

Figure S6. Effects of substitutions introduced in the 910S-ACCU substrate in selected positions outside the central tetranucleotide on cleavage efficiency. For each enzyme, the cleavage efficiency was normalized to the cleavage efficiency of the wildtype 910S-ACCU substrate. dsRNA (0.1 µg) was digested with 80 ng of CkMiniIII, 5.5 µg of CrMiniIII, 80 ng of TmMiniIII, and 7 ng of TtMiniIII. The reactions with CkMiniIII and TmMiniIII were performed for 30 min. The reactions with TtMiniIII were performed for 60 min. The reactions with CrMiniIII were performed for 90 min.

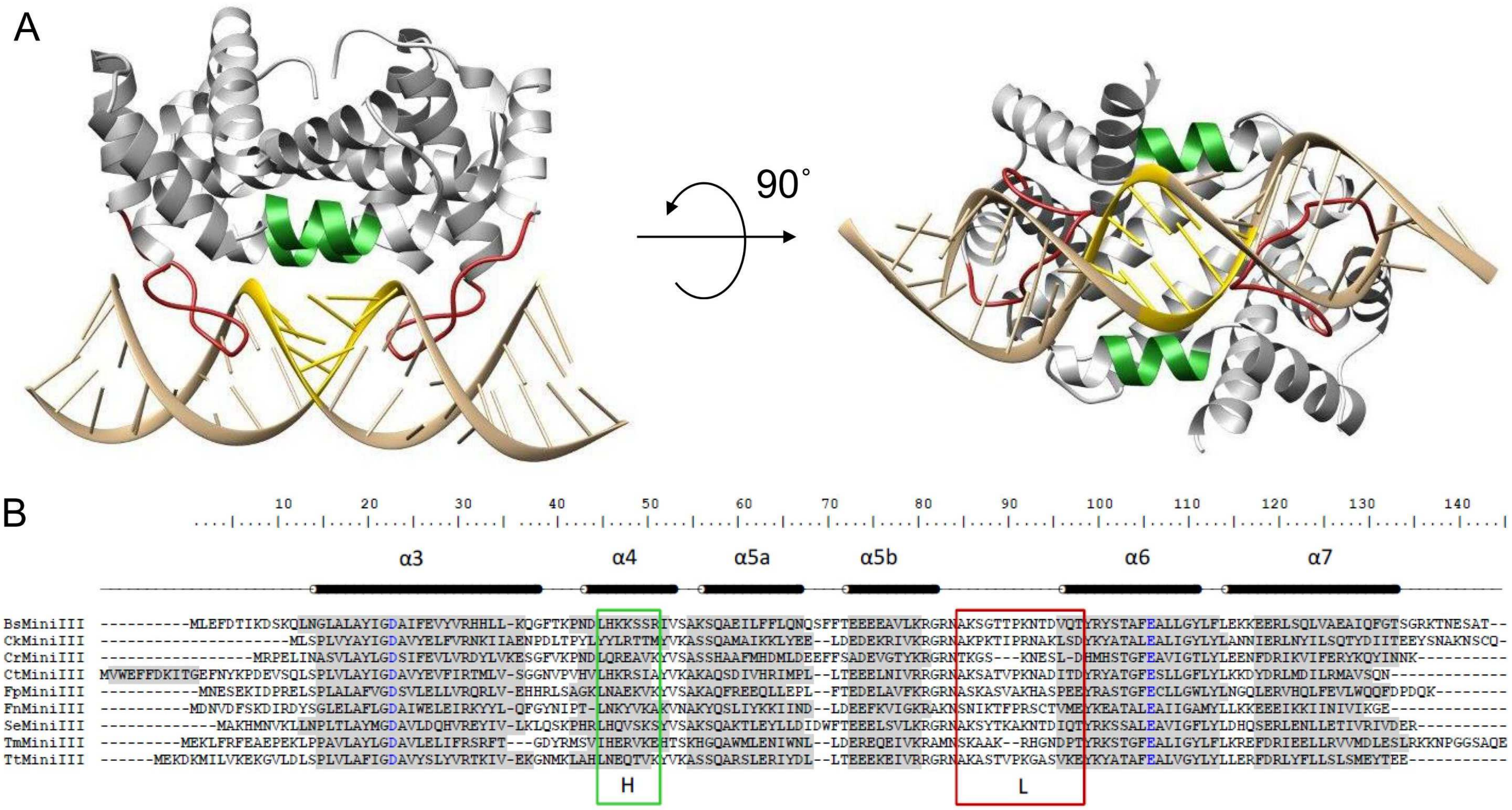

Figure S7. Structural elements (α4 helix and α5b-α6 loop) within the context of the BsMiniIII structure and Mini-III protein sequence alignment. (A) Theoretical model of the BsMiniIII structure bound to dsRNA[17]. The central tetranucleotide of the target dsRNA is shown in yellow. The extent of the α4 helix and α5b-α6 loop sequences that were replaced in the swapping experiment is shown in green and red, respectively. (B) Alignment of Mini-III sequences from selected organisms. The numeration of the residues and secondary structures of Mini-III from *B. subtilis* are shown on top (cylinders represent α-helices). Conserved residues of the active site are shown in blue (D23 and E 106 in BsMiniIII). The sequences of the α4 helix (H) and α5b-α6 loop (L) that were replaced in the swapping experiment are marked by green and red frames respectively, and localization of predicted α helices is shown by gray background.

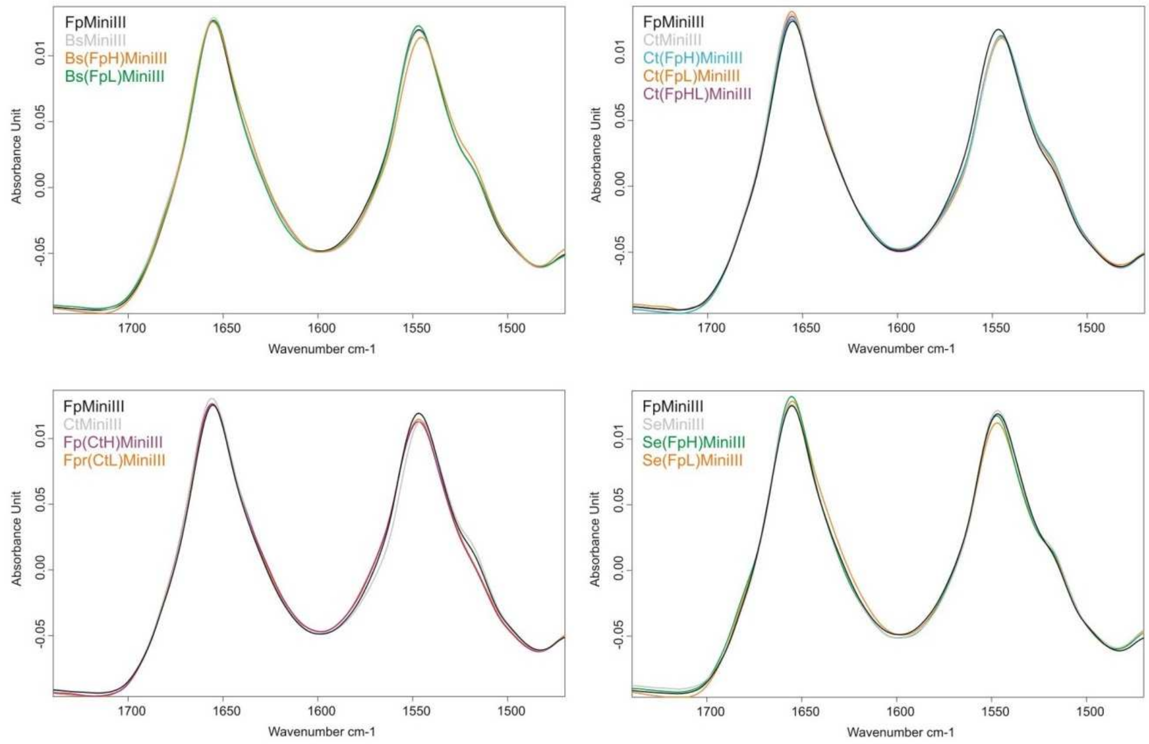

Figure S8. Comparison of amide I – amide II region of FT-IR spectra of swap variants and donor and acceptor proteins. Buffer of the protein samples was exchanged to 150 mM NaCl, 50 mM HEPES pH 7.5 by three cycles of diafiltration in Microcon ultrafiltration devices (Merck Millipore), then samples were diluted with the same buffer to concentration of 2 mg/ml and FT-IR spectra were measured in BioATRCell II on Tensor 27 FT-IR spectrometer (Bruker). All spectra were vector normalized in the presented range. There is no visible differences in amide I peaks shapes that would indicate substantial differences in the secondary structure content between compared proteins.

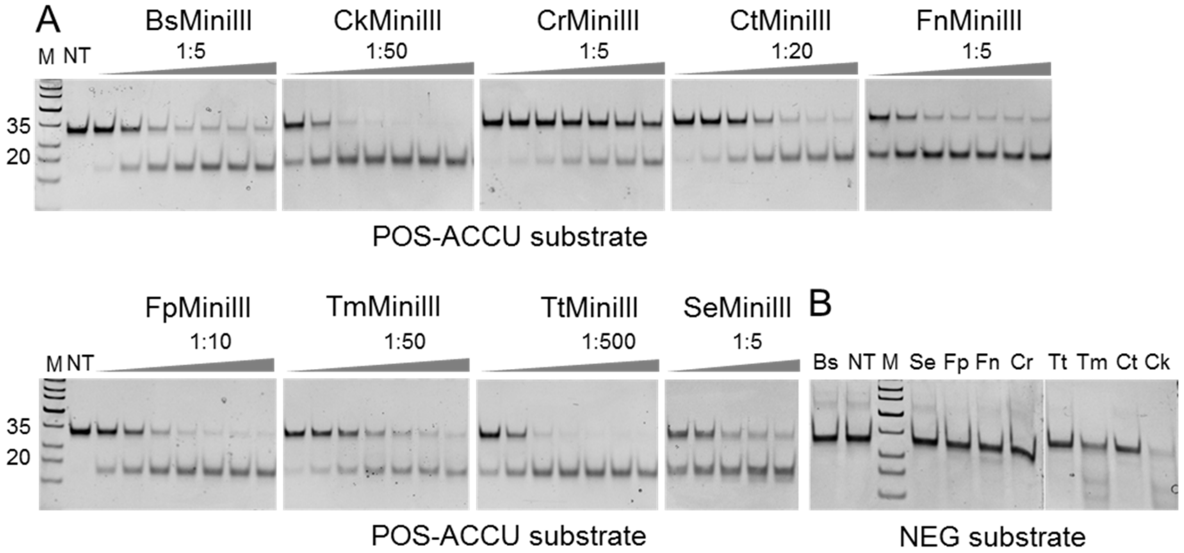

Figure S9. Cleavage of dsRNA oligonucleotides in multiple turnover reaction. Enzyme:substrate stoichiometries in reactions are indicated above each assay. For POS-ACCU substrate aliquots were taken after 0.25, 0.5, 1, 2, 3, 4 and 5 hours of reaction except for SeMiniIII, where reaction times were 18, 24, 48, 72 and 93 hours. For NEG substrate where the same enzyme:substrate stoichiometries were used as for POS-ACCU only samples obtained after longest reaction times are presented, i.e after 5 hours except for SeMiniIII where reaction time was 93 hours. NT – untreated substrate; M – molecular weight marker (GeneRuler Ultra Low Range DNA Ladder, Thermo).

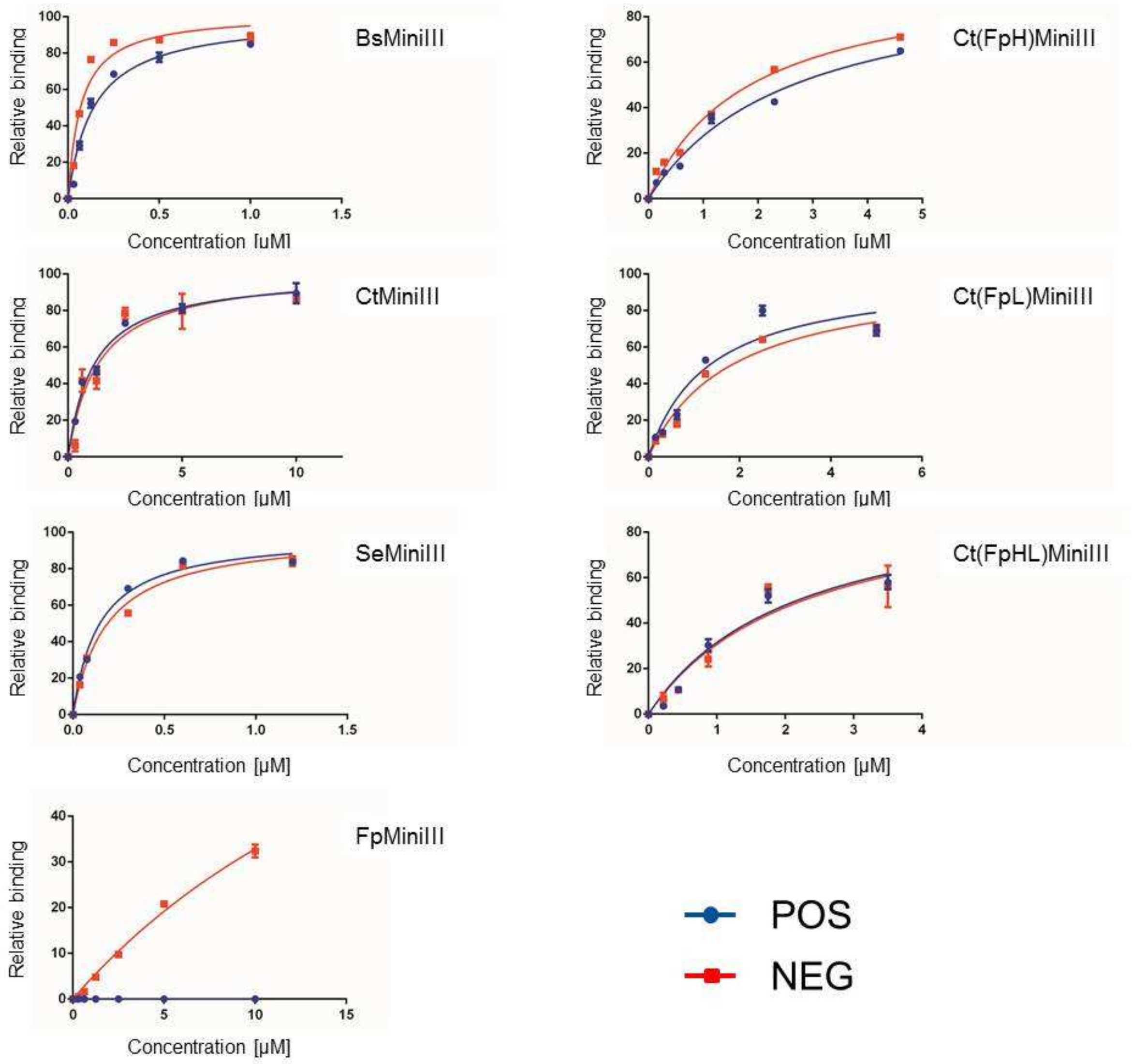

Figure S10. Binding isocrates for dsRNAs with (POS-ACCU) and without (NEG) preferred cleavage site.

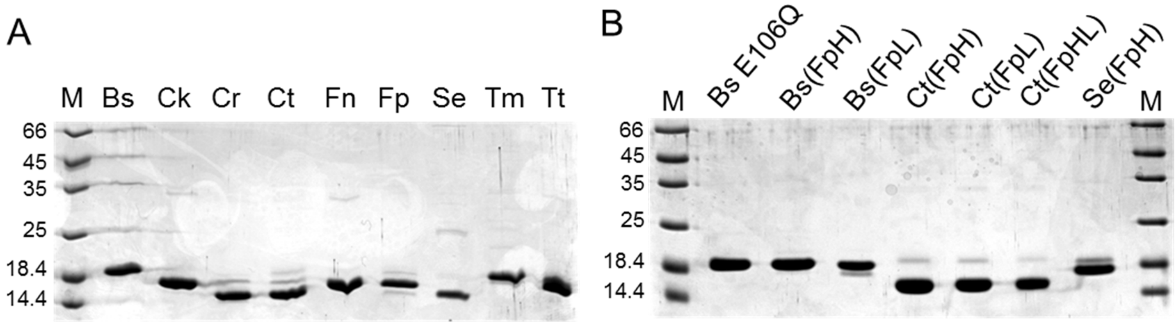

Figure S11. Purified Mini-III enzymes. 1 μg aliquotes of the purified Mini-IIIs were resolved in 15% SDS-PAGE and stained with Commassie Brilliant Blue. A – wt proteins; B – BsMiniIII E106Q and swap variants. M – molecular weight standard; molecular masses (in kDa) shown.

Table S1. Reaction buffers tested in the cleavage optimization step.

Buffer	Formula
B	10 mM Tris-HCl pH 7.5, 10 mM $MgCl_2$, 0.1 mg/mL BSA
B1	10 mM Tris-HCl pH 7.5, 1 mM $MgCl_2$, 0.1 mg/mL BSA
G	10 mM Tris-HCl pH 7.5, 10 mM $MgCl_2$, 50 mM NaCl, 0.1 mg/mL BSA
G1	10 mM Tris-HCl pH 7.5, 1 mM $MgCl_2$, 50 mM NaCl, 0.1 mg/mL BSA
O	50 mM Tris-HCl pH 7.5, 10 mM $MgCl_2$, 100 mM NaCl, 0.1 mg/mL BSA
O1	50 mM Tris-HCl pH 7.5, 1 mM $MgCl_2$, 100 mM NaCl, 0.1 mg/mL BSA
R	10 mM Tris-HCl pH 8.5, 10 mM $MgCl_2$, 100 mM KCl, 0.1 mg/mL BSA
R1	10 mM Tris-HCl pH 8.5, 1 mM $MgCl_2$, 100 mM KCl, 0.1 mg/mL BSA
Y	33 mM Tris-acetate pH 7.9, 20 mM magnesium acetate, 66 mM potassium acetate, 0.1 mg/mL BSA
2XY	66 mM Tris-acetate pH 7.9, 40 mM magnesium acetate, 132 mM potassium acetate, 0.2 mg/mL BSA
Bs	10 mM Tris-HCl pH 7.5, 1 mM $MgCl_2$, 5 mM NaCl, 0.1 mg/mL BSA
BG	10 mM Tris-HCl pH 7.5, 10 mM $MgCl_2$, 25 mM NaCl, 0.1 mg/mL BSA

Table S2. Fragments of Φ6 dsRNA used as substrates in cleavage and binding assays.

Substrate name	Segment	Sequence coordinates	Cleavage position	Substrate size (bp)	Sequence
910	S	804-948	910	156	GGGAA**<u>ACCU</u>**CUCUC[1]
949	S	910-998	949	100	CAAAC**<u>ACGA</u>**GUUCG
2021	L	1786-2112	2021	338	UCGAG**<u>ACCU</u>**CGCUC
3292	L	3041-33884	3292	355	AGGGU**<u>AUCU</u>**ACUCU
4486	L	4421-4569	4486	160	CCGCU**<u>ACGU</u>**ACCCC
4754	L	4650-4928	4754	290	GAAGG**<u>AGCU</u>**CGACC
POS-ACCU	S	897-924	910	28	GGGAA**<u>ACCU</u>**CUCUC
NEG	S	822-849	-	28	-

[1]The central tetranucleotide of the preferred cleavage site is shown in bold and underlined.

Table S3. Secondary structure analysis of Mini-III enzymes with FT-IR.

Protein	α-helices [%]		β-sheets [%]	
	PSIPRED[1]	FT-IR[2]	PSIPRED	FT-IR
BsMiniIII structure[3]	51.7		0	
BsMiniIII	54.6	52.3	0	7.2
Bs(FpH)MiniIII		50.7		9.1
Bs(FpL)MiniIII		54.3		6.7
BsMiniIII E106Q		54.0		3.9
CkMiniIII	57.9	53.4	0	4.8
CrMiniIII	59.3	57.0	0	5.2
CtMiniIII	58.1	53.6	0	6.5
Ct(FpH)MiniIII		51.8		7.6
Ct(FpL)MiniIII		54.1		6.6
Ct(FpHL)MiniIII		52.8		7.4
FnMiniIII	56.3	53.3	0	5.4
FpMiniIII	59.1	53.3	0	7.5
Fp(CtH)MiniIII		52.6		8.4
Fp(CtL)MiniIII		52.4		7.8
SeMiniIII	64.3	55.4	0	6.7
Se(FpH)MiniIII		53.3		5.8
Se(FpH)MiniIII		52.0		10.2
TmMiniIII	55.0	51.2	0	7.8
TtMiniIII	55.8	55.1	0	5.3

Secondary structure content analysis was done with OPUS 6.5 software (Bruker) using FT-IR spectra shown in Supplementary Figure S9. [1] Fraction of a sequence forming secondary structure according to PSIPRED prediction. [2] Fraction of a sequence forming secondary structure according to FT-IR spectrum analysis. [3] Fraction of a sequence forming secondary structure according to the crystal structure of BsMiniIII (PDB code 4OUN). Majority of values of α-helices quantity obtained from FT-IR do not deviate from PSIPRED estimations by more than 5%. There are also no significant differences between secondary structures of swap variants and original proteins suggesting no destabilization of the variants.

Table S4. Primers used for cloning Mini-III and L3 ORFs in expression vectors.

**Primer name**	**Primer sequence**
FckminiIII	CCTCATATGGTCAGTCCTTTAGTATATG
RckminiIII	CCTCTCGAGTTATTGACAGCTATTCTTGGC
FcrminiIII	GGACATATGGGCCCTGAACTGATTAATGC
RcrminiIII	GGCCTCGAGTTATTTGTTGTTGATGTACTG

FctminiIII	CAGGCATATGGTTTGGGAATTTTTTGAC
RctminiIII	GACCTCGAGTCAATTCTGTGAAACAGCC
FfnminiIII	CCGCATATGGACAATGTAGATTTTTCAAAG
RfnminiIII	GTGCTCGAGTCATCATTCTCCCTTTATAACTATATTTATAATTTTTT TTATTTC
FfpminiIII	GGACATATGGACGAAAGCGAAAAAATTG
RfpminiIII	GCGCTCGAGTTATTTCTGATCAGGATCAAAC
FseminiIII	TAGACATATGGCAGTGGCTAAACATATGAAC
RseminiIII	ATCTCGAGCTACCTTTCATCCACTA
FtmminiIII	GCTTCATATGGAAAAACTCTTCAGATTCG
RtmminiIII	CTTCTCGAGTTATTCCTGAGCGCTTCC
FttminiIII	CGCACATATGGAAAAGGATAAGATGATTCTTG
RttminiIII	GCTCTCGAGTCATTCTTCCGTGTATTCCATAG
fRL3	CACCATATGACCAAAGGAATCTTAG
rRL3	GCCTCGAGTTTAGATTTAACAGCACTTTT
E106Qfv[1]	GGCGCTTCTGGGCTACCTTTTTC
E106Qrev[1]	TGAAATGCTGTACTGTAGCGGTAC

Restriction sites used for cloning are underlined.[1] Primers used to introduce E106Q substitution in BsMiniIII

Table S5. Primers used to create constructs that encoded chimeric Mini-III.

**Primer name**	**Primer sequence**
Amplification of the construct backbone	
CpR1f	TATGTCAAAGCAAAGGCAC
CpR1r	TCAGAACATGTACCGGTACG
CpR2f	TACAGGTATGCTACCGGTTTTGAGTCTTTG
CpR2r	CGTTCCTTCCCCTGCGGAC
FpR1f	TACGTTAGCGCCAAAG
FpR1r	TTACCTGCGCTCAGAC
FpR2f	TATCGTGCAAGCACCGGTTTTG

FpR2r	CGACCACGTTTAAAAACTGCCAGTTC
BsR1f	TATGTTTCAGCAAAGTCACA
BsR1r	TCAGATCATTTGGTTTGGTAAAG
BsR2f	TACCGCTACAGTACAGC
BsR2r	CGTTTCTGCCTCTTTTCAGC
SeR1f	TACGTTTCAGCGAAAAGTC
SeR1f	TCAGACGATGAGGTTTACTTTGTAATTTTAG
SeR2f	TATCGTAAAAGTTCAGCGTTAG
SeR2r	CGTTACGTCCTCGTTTTAAAAC
Primers for creating inserts	
CtR1Up	GCTTCATAAGCGCTCCATTGCT
CtR1Dw	AGCAATGGAGCGCTTATGAAGC
CtR2Up	CAATGCCAAATCGGCCACGGTTCCGAAAAATG
CtR2Dw	ATCCGTAATATCCGCATTTTTCGGAAC
FpR1Up	ATGCAGAAAAAGTTAAA
FpR1Dw	TTTAACTTTTTCTGCAT
FpR2Up	CGTCAAAAGCAAGCGTTGCAAAACATG
FpR2Dw	TTCTTCCGGACTTGCATGTTTTGCAAC

Table S6. Primers used to create dsRNA substrates for cleavage and binding assays.

**Primer name**	**Primer sequence**
Primers used in RT-PCR to create templates for dsRNA synthesis	
910fT7	TAATACGACTCACTATAGGGCTGCTCGCGCGTTG[1]
910rP6	GGAAAAAAATCAGACACAACTGACGCGATCG
949fT7	TAATACGACTCACTATAGGGCCTCTCTCTCTGGCCACGATC
949rP6	GGAAAAAAATGCCCTGTACAGCAGGCATAAG
2021fT7	TAATACGACTCACTATAGGGCTCCTATCATGGCCGTTGC
2021rP6	GGAAAAAAACTTCGAGATCAGGGTTGGACG
3292fT7	TAATACGACTCACTATAGGGTACCGCGATCAACACTGTCGTC

3292rP6	GGAAAAAAACGAATCAGGACGTCTGGACG
4486fT7	TAATACGACTCACTATAGGGCTGTCTCCCCTCGGTTTCATC
4486rP6	GGAAAAAAATCGACAGACGACAGCGCTG
4754fT7	TAATACGACTCACTATAGGGCTCATCGCCTCGATGAACCAAG
4754rP6	GGAAAAAAACTACTGCTTTCGAGCGGTCG
dsRNA oligonucleotides used for binding assays and multiple turnover cleavage assays[2]	
NEG	CCUGUCCCCCGCCGAAGUCGCUGCAAUU
POS-ACCU	GCUGGGAGGGAAACCUCUCUCUCUGGCC
POS-UGCA	GCUGGGAGGGAAUGCACUCUCUCUGGCC

[1]Sequences of T7 RNA polymerase and Φ6 RNA polymerase are underlined, and the first transcribed nucleotide is shown in bold. [2]The sequence of one strand is shown.

Table S7. Primers used to introduce substitutions into cleavage site in 910S substrate.

Primer name	Primer sequence
WSSWf	SSWCTCTCTCTGGCCACGATC
WSSWr	WTTCCCTCCCAGCACG
ANNTf	NNTCTCTCTCTGGCCACGATC
ANNTr	TTTCCCTCCCAGCACG
NCCNf	CCNCTCTCTCTGGCCACGATC
NCCNr	NTTCCCTCCCAGCACG
